# Osteology and phylogeny of *Robustichthys luopingensis*, the largest holostean fish in the Middle Triassic

**DOI:** 10.7717/peerj.7184

**Published:** 2019-06-24

**Authors:** Guang-Hui Xu

**Affiliations:** 1 Key Laboratory of Vertebrate Evolution and Human Origins of Chinese Academy of Sciences, Institute of Vertebrate Paleontology and Paleoanthropology, Chinese Academy of Sciences, Beijing, China; 2 CAS Center for Excellence in Life and Paleoenvironment, Beijing, China

**Keywords:** Fossils, Holostei, Halecomorphi, Ionoscopiformes, Evolution

## Abstract

The extinct ray-finned fish taxon *Robustichthys luopingensis* from Luoping, eastern Yunnan, China represents the largest holostean known in the Middle Triassic. Despite its potential significance for investigating the holostean phylogeny and reconstructing the Triassic marine ecosystems, *Robustichthys* has so far not been described in detail and its phylogenetic position within the Holostei was controversy. This study provides a redescription and revision of *Robustichthys* based upon a comparative study of eight type specimens and nine new specimens. Newly recognized information includes a toothed parasphenoid, a pair of premaxillae not pierced by the olfactory nerve, a splint-like quadratojugal, a hatchet-shaped hyomandibula, an hourglass-shaped symplectic, anterior and posterior ceratohyals, a complete series of branchiostegal rays, and sclerotic bones. A revised reconstruction of *Robustichthys* is presented. Results of a cladistic analysis confirmed *Robustichthys* as an ionoscopiform within the Halecomorphi; the previous placements of *Robustichthys* as a basal ginglymodian and Ionoscopidae as a basal amiiform clade are not supported. The sister group relationship between Sinamiinae (*Sinamia* and *Ikechaoamia*) and Amiinae (*Amia* and *Cyclurus*) within the Amiidae is newly recognized. This revised topology provides new insights into the evolution and historical paleoecology of halecomorph fishes.

## Introduction

The Neopterygii are a highly speciose group of ray-finned fishes with three major clades usually recognized, Ginglymodi (lepisosteids or gars and their relatives), Halecomorphi (*Amia* or bowfin and their relatives), and Teleostei (the largest radiation of aquatic vertebrates). The interrelationships of these three neopterygian clades were previously one of the greatest challenges of vertebrate systematics (Sallan, 2014; Friedman, 2015). The earliest morphological studies supported a monophyletic Holostei containing lepisosteids and *Amia* (Huxley, 1861; Regan, 1923; Goodrich, 1930; Romer, 1945; Nelson, 1969). Gardiner (1960) first considered that *Amia* and teleosts might be descended from a common ancestor not shared with gars. This hypothesis, more clearly expressed by Patterson (1973), was later widely accepted by many authors (Rosen et al., 1981; Grande & Bemis, 1998; Liem et al., 2001). It was not until the last decade that morphological and molecular data reached a consensus on the sister group relationship between Halecomorphi and Ginglymodi and, consequently, the Holostei concept was resurrected (Hurley et al., 2007; Grande, 2010; Nakatani et al., 2011; Xu & Wu, 2012; Near et al., 2012; Broughton et al., 2013; Cavin, Deesri & Suteethorn, 2013; Giles et al., 2017; Sun et al., 2017; López-Arbarello & Sferco, 2018; López-Arbarello et al., 2016, 2019; Xu, Ma & Ren, 2018a; Xu, Ma & Zhao, 2018b; Xu et al., 2019).

However, the deep evolutionary history of Holostei remain obscure, partly because of insufficient studies of fossil records. Pre-Permian fossil evidence of holosteans has never been reported, although molecular analyses (Azuma et al., 2008; Inoue et al., 2009; Setiamarga et al., 2009; Nakatani et al., 2011) estimate the Holostei/Teleostei split within the Neopterygii to be between 362 and 365 Ma (Famennian, Late Devonian). The late Permian (Wuchiapingian) “semionotid”-like taxon *Acentrophorus* promisingly represents the earliest holostean, but urgently needs restudy and formal analysis (Gill, 1923; Gardiner, 1960; Friedman, 2015). The earliest halecomorphs and ginglymodians are represented by the Early Triassic parasemionotiforms (Olsen, 1984; Grande & Bemis, 1998) and the early Middle Triassic (Anisian) kyphosichthyiforms (Xu et al., 2019), respectively. Notably, the sister group of Holostei, Teleostei, was first known in the late Middle Triassic (Ladinian) (Arratia, 2013; Tintori et al., 2015). The holosteans, outstripped the coeval teleosts in terms of taxonomic diversity in the Middle Triassic (Clarke & Friedman, 2018), are particularly significant for understanding the early evolutionary history of Neopterygii.

Up to date, 14 holostean species (in 13 genera) have been reported from a series of well-preserved fossil assemblages from the Middle Triassic marine rock succession in Southwest China, including seven in the Anisian Luoping biota (Tintori et al., 2010; López-Arbarello et al., 2011; Xu & Wu, 2012; Wen et al., 2012; Xu, Zhao & Coates, 2014; Xu et al., 2019; Ma & Xu, 2017), two in the Anisian Panxian biota (Chen et al., 2014; Xu & Shen, 2015), and five in the Ladinian Xingyi biota (Su, 1959; Liu, Yin & Wang, 2002; Liu et al., 2003; Xu & Ma, 2018; Xu, Ma & Ren, 2018a). Among them, most holosteans from the Xingyi biota were known as early as 60 years ago but many were incompletely described. *Asialepidotus*, originally considered as a semionotid ginglymodian (Su, 1959), has recently been revised as an ionoscopiform halecomorph (Xu & Ma, 2018). Three other holosteans from this biota, *Sinoeugnathus* (Su, 1959), *Guizhouamia* (Liu, Yin & Wang, 2002) and *Xingyia* (Liu et al., 2003), although placed in the Amiiformes, lack formal phylogenetic analyses and need further studies. The recently reported kyphosichthyiform *Fuyuanichthys* documents the first ginglymodian known in the Xingyi biota (Xu, Ma & Ren, 2018a). All seven holosteans from the Luoping biota were named in the last decade but received more attention than those from the Xingyi biota because of their superb preservation and older age, including three kyphosichthyiform ginglymodians (*Lashanichthys sui*, López-Arbarello et al., 2011; *Kyphosichthys*, Xu & Wu, 2012; *Yudaiichthys*, Xu et al., 2019), two ionoscopiform halecomorphs (*Robustichthys*, Xu et al., 2014; *Subortichthys*, Ma & Xu, 2017) and two specialized, taxonomically controversial holosteans (the naked *Gymnoichthys*, Tintori et al., 2010; and the deep-bodied *Luoxiongichthys*, Wen et al., 2012). Holosteans from the Panxian biota are represented by relatively few specimens and their studies are still on the initial stage, with only a single kyphosichthyiform ginglymodian (*Lashanichthys yangjuanensis*, Chen et al., 2014) and an ionoscopiform halecomorph (*Panxianichthys*, Xu & Shen, 2015) reported in the last 5 years.

**Figure 1:**
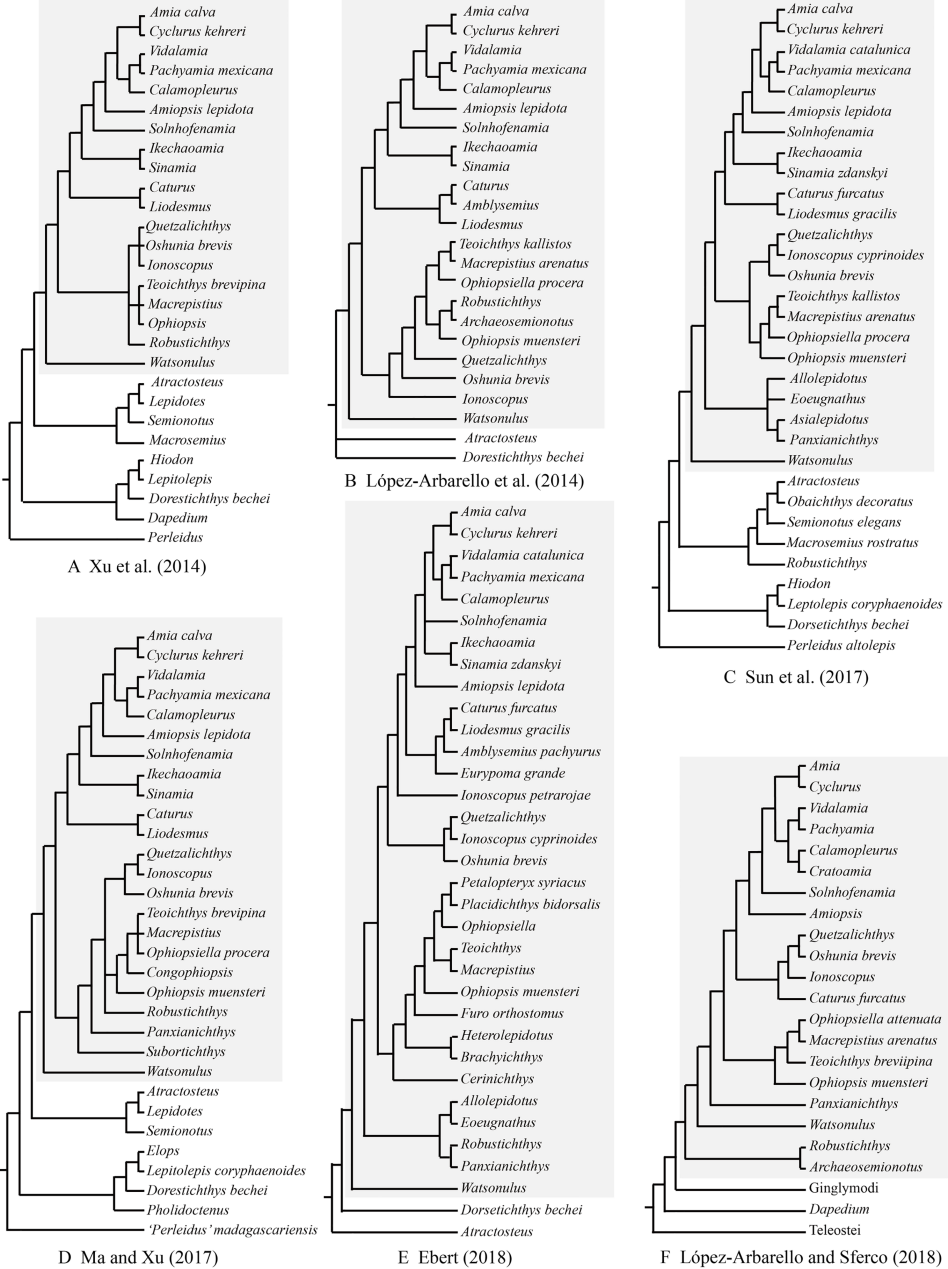
Selected previous hypotheses of phylogenetic relationships of *Robustichthys*. Trees showing previous hypotheses of (A) Xu, Zhao & Coates (2014), (B) López-Arbarello, Stockar & Bürgin (2014), (C) Sun et al. (2017), (D) Ma & Xu (2017), (E) Ebert (2018) and (F) López-Arbarello & Sferco (2018). Halecomorphi have been highlighted in each cladogram with a gray rectangle.

As far as I have known, *Robustichthys* represents the largest holostean in the Middle Triassic with a maximum standard length (SL) up to 360 mm (IVPP V20414). The second and third largest holosteans in this epoch are the halecomorph *Asialepidotus* (Su, 1959; Xu & Ma, 2018) and the ginglymodian *Ticinolepis* (López-Arbarello et al., 2016), which have a maximum SL of 273 and 250 mm, respectively; other Middle Triassic holosteans generally have a maximum SL no larger than 180 mm. Because of its large size and high quality of preservation, *Robustichthys* potentially provides important information on the morphological diversification of early holosteans and the reconstruction of Triassic marine ecosystems. However, this taxon has not been described in detail since it was named in 2014. Some character states were unknown or improperly coded for *Robustichthys* in several previous phylogenetic analyses, which resulted in controversies on its phylogenetic position within the Holostei (Fig. 1). *Robustichthys* was recovered as a basal ionoscopiform by Xu and his colleagues (Xu, Zhao & Coates, 2014; Xu et al., 2019; Xu & Shen, 2015; Ma & Xu, 2017; Xu & Ma, 2018), as a “furid” ionoscopiform or a basal halecomorph by López-Arbarello and her colleagues (López-Arbarello, Stockar & Bürgin, 2014; López-Arbarello & Sferco, 2018), as a basal ginglymodian by Sun and his colleagues (Sun et al., 2017; Sun & Ni, 2018), and as a “panxianichthyiform” halecomorph by Ebert (2018).

The aim of this study is to provide a detailed description and revision of the osteology of *Robustichthys* and a comprehensive and up-to-dated discussion on the phylogenetic relationships of this taxon with other holosteans. It is hoped that the present work may contribute for a better understanding the comparative anatomy, evolution and phylogeny of the Holostei in general.

## Material and Methods

Most specimens are curated at the fossil collections of the Institute of Vertebrate Paleontology and Paleoanthropology (IVPP), Chinese Academy of Sciences, with two at those of the Zhejiang Museum of Natural History, Hangzhou, China. All specimens were mechanically prepared with sharp steel needles. Illustrations were drawn manually and then prepared using Adobe Photoshop and Illustrator software packages (CS5). The relative position of fins and scale counts were expressed following Westoll (1944). The actinopterygian nomenclature was utilized following Grande & Bemis (1998) and Grande (2010). The phylogenetic framework for the discussions provided in the present paper is based on the results of a cladistic analysis of neopteryigan phylogeny including 224 morphological characters and 60 extant and fossil terminal taxa. The characters were mainly adopted or modified from previous analyses of neopterygian phylogeny (Gardiner & Schaeffer, 1989; Olsen & McCune, 1991; Gardiner, Maisey & Littlewood, 1996; Grande & Bemis, 1998; Coates, 1999; Arratia, 1999, 2013; Cavin & Suteethorn, 2006; Alvarado-Ortega & Espinosa-Arrubarrena, 2008; Cavin, 2010; Grande, 2010; Xu & Gao, 2011; Xu et al., 2012, 2019; Xu, Zhao & Coates, 2014; Xu, Gao & Coates, 2015; Xu, Ma & Ren, 2018a; Xu, Ma & Zhao, 2018b; Xu & Ma, 2016; Xu & Zhao, 2016; López-Arbarello, 2012; Brito & Alvarado-Ortega, 2013; Cavin, Deesri & Suteethorn, 2013; Deesri et al., 2014; Deesri, Jintasakul & Cavin, 2016; Giles et al., 2017; Ebert, 2018; López-Arbarello & Sferco, 2018). All characters were unordered and equally weighted. *Pteronisculus stensioi* was selected for out-group comparison. Tree searches were accomplished with the heuristic search algorithm (gaps treated as missing data; 10,000 random addition sequence replicates; tree bisection-reconnection branch-swapping, with 10 trees held at each step and multiple trees saved) in PAUP* 4.0b10 (Swofford, 2003).

## Results

### Systematic palaeontology

Actinopterygii Cope, 1887
Neopterygii Regan, 1923
Holostei Müller, 1844
Halecomorphi Cope, 1872
Ionoscopiformes Grande and Bemis, 1998
*Robustichthys luopingensis* Xu et al., 2014
(Figs. 2–11)

**Figure 2:**
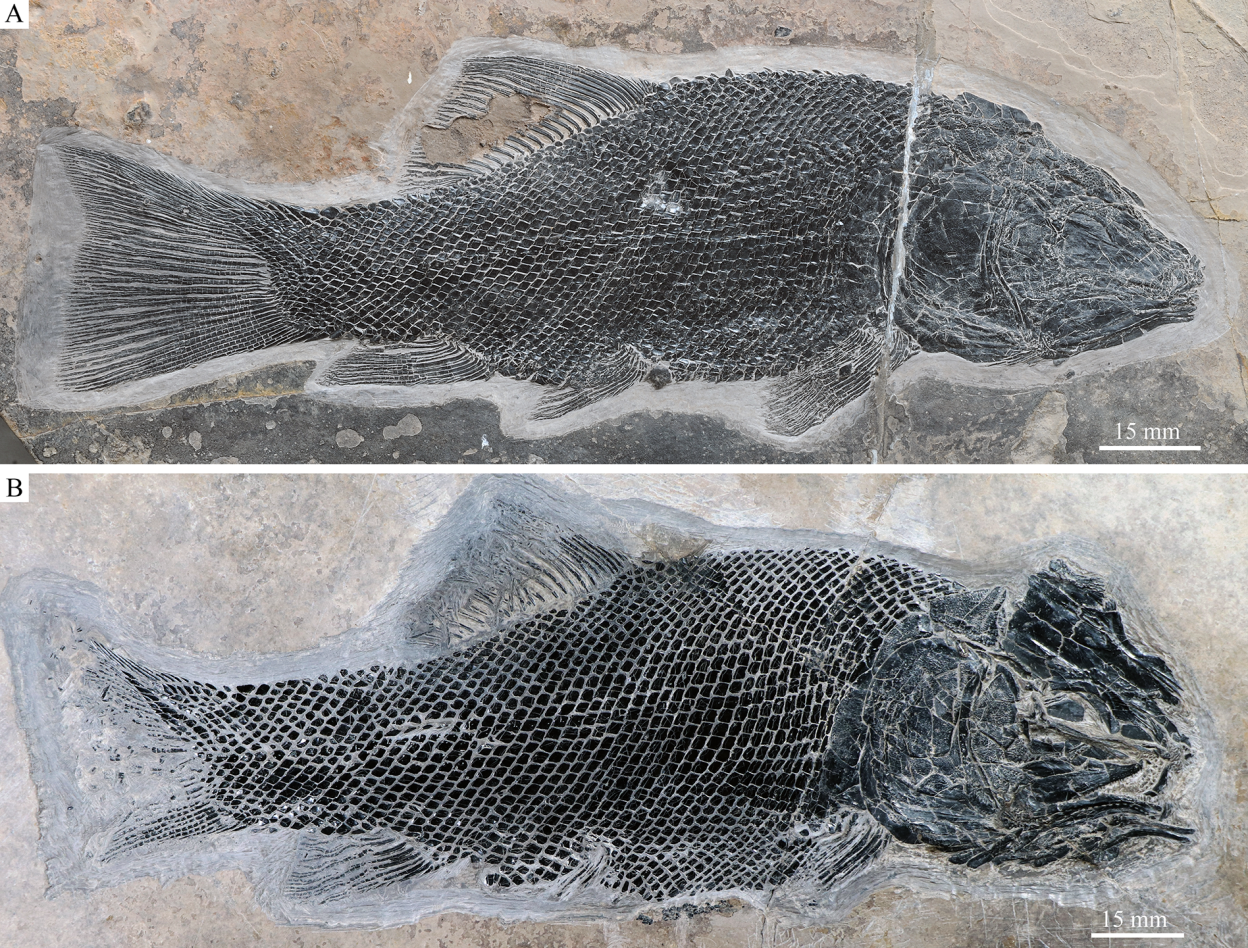
IVPP V18568 (holotype) and V20416. *Robustichthys luopingensis*. (A) IVPP V18568 (holotype). (B) IVPP V20416.

**Figure 3:**
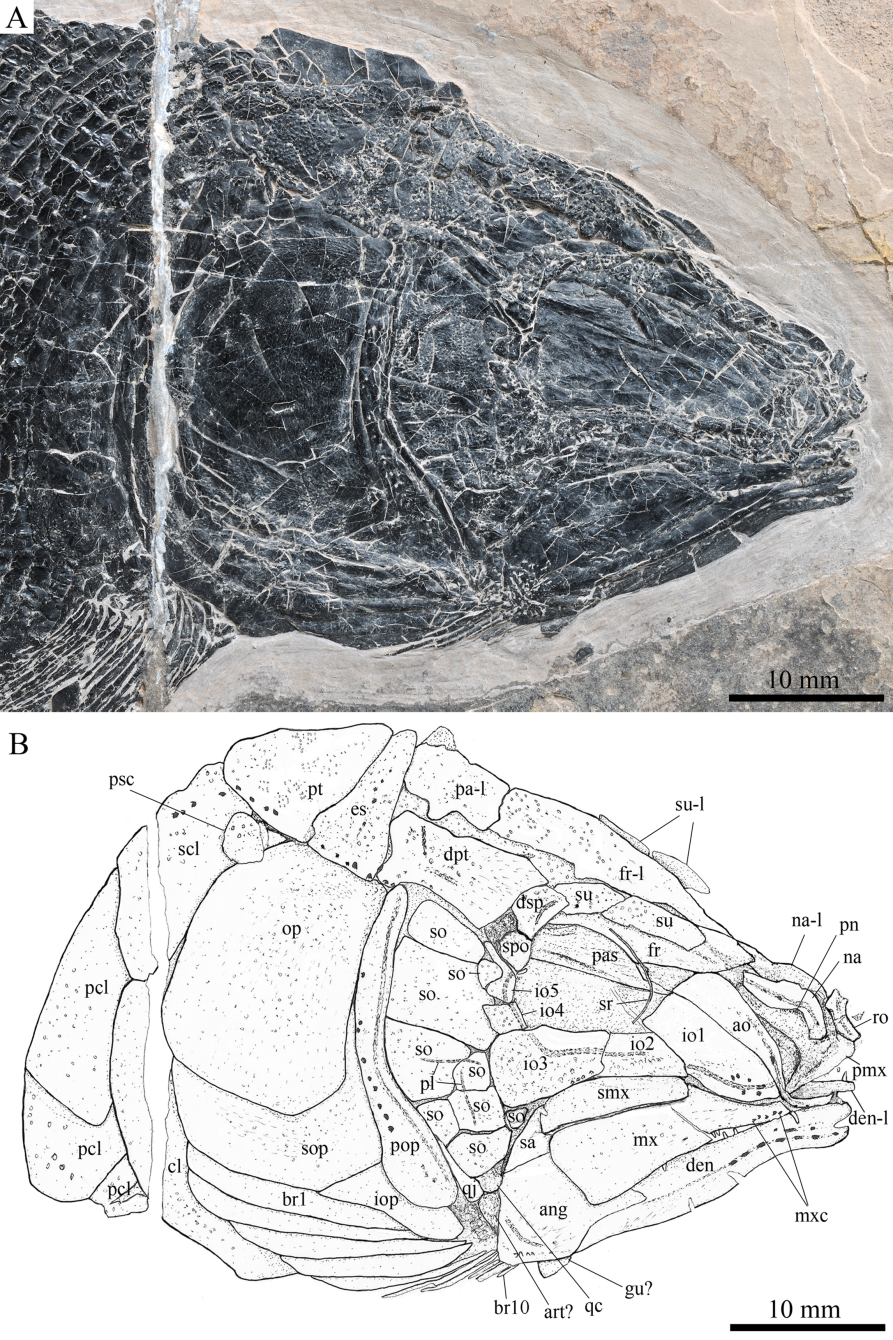
Skull and pectoral girdle in the holotype. Skull and pectoral girdle of *Robustichthys luopingensis*, IVPP V18568 (holotype). (A) Photograph. (B) Line drawing.

**Figure 4:**
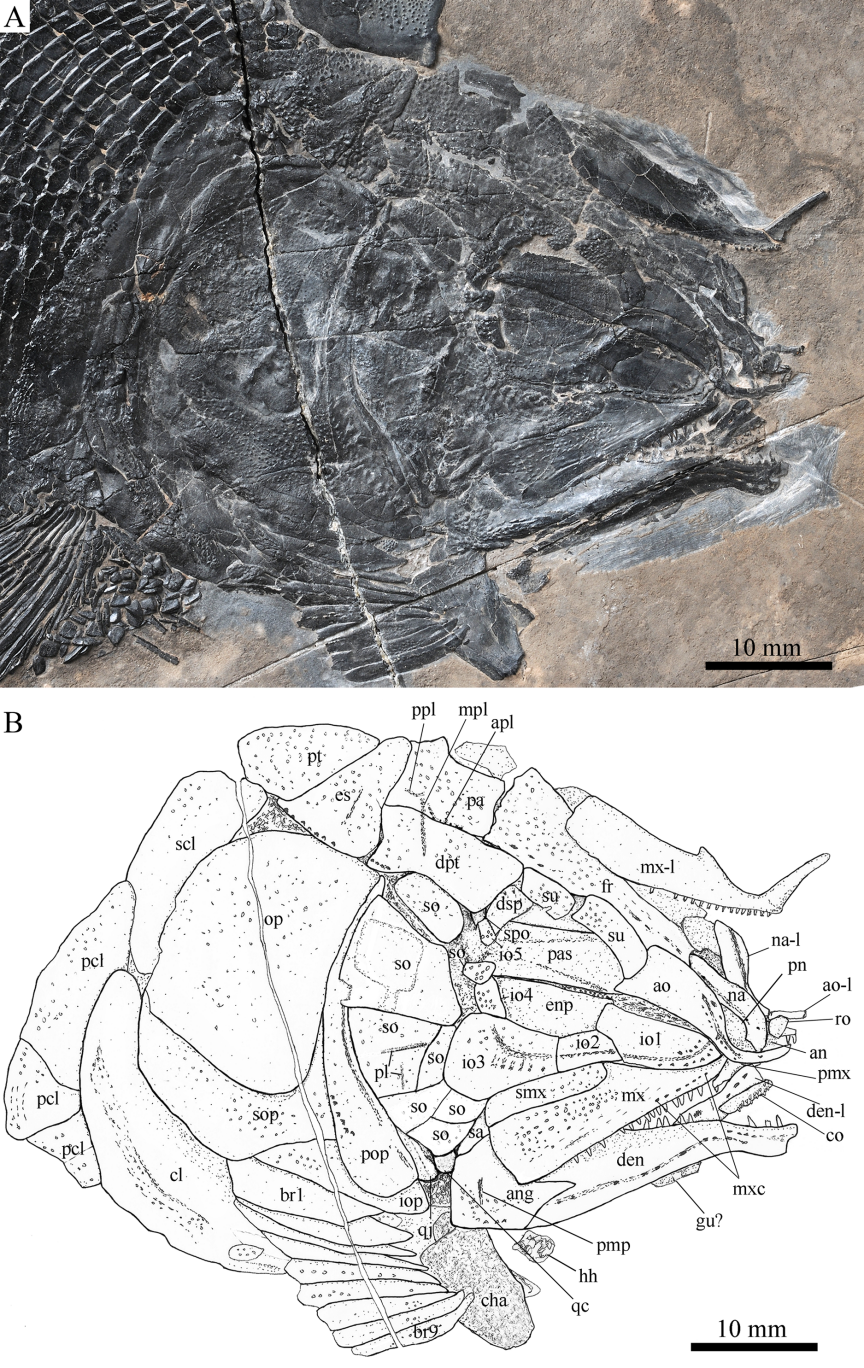
Skull and pectoral girdle in ZMNH M1690. Skull and pectoral girdle of *Robustichthys luopingensis*, ZMNH M1690. (A) Photograph. (B) Line drawing.

**Figure 5:**
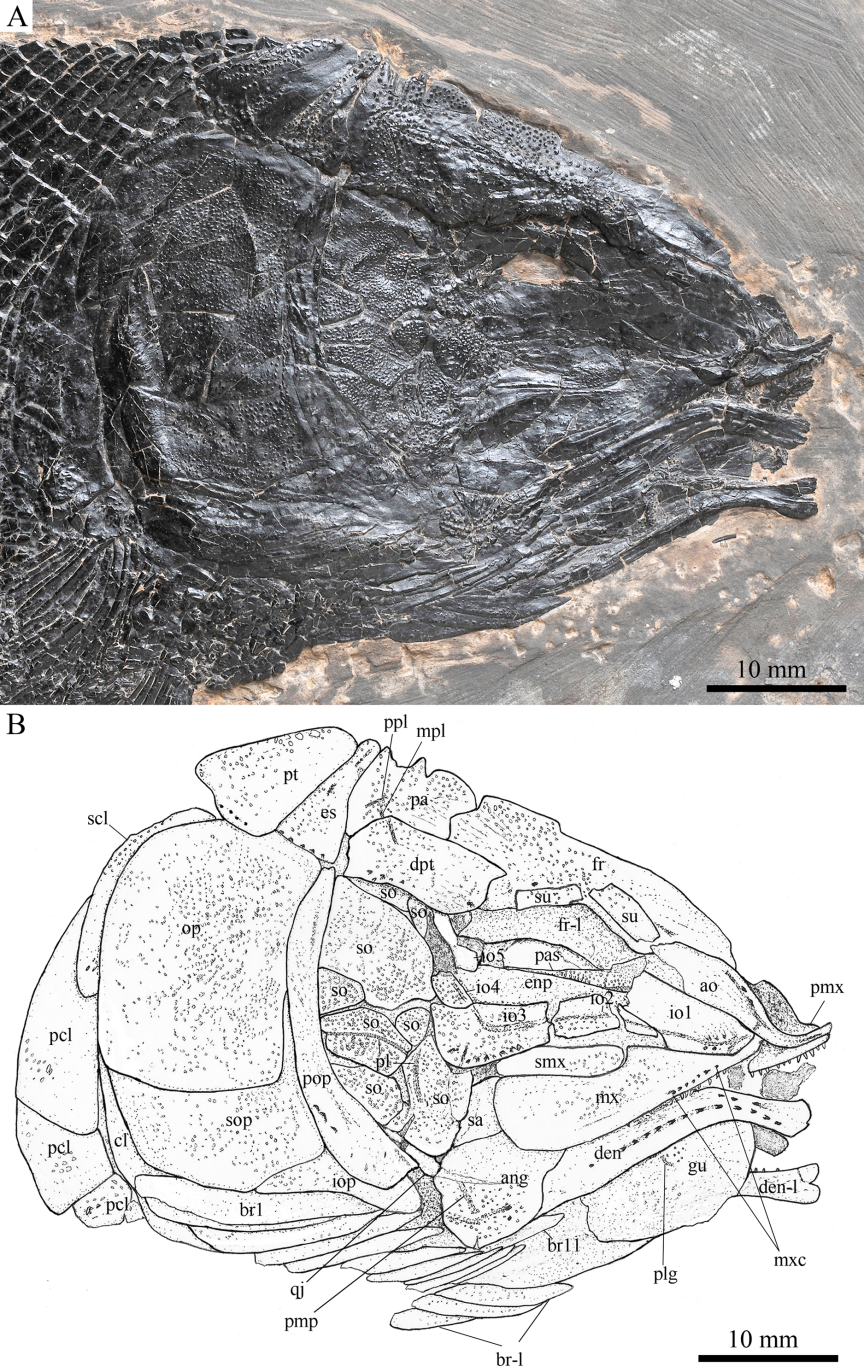
Skull and pectoral girdle in ZMNH M1691. Skull and pectoral girdle of *Robustichthys luopingensis*, ZMNH M1691. (A) Photograph. (B) Line drawing.

**Figure 6:**
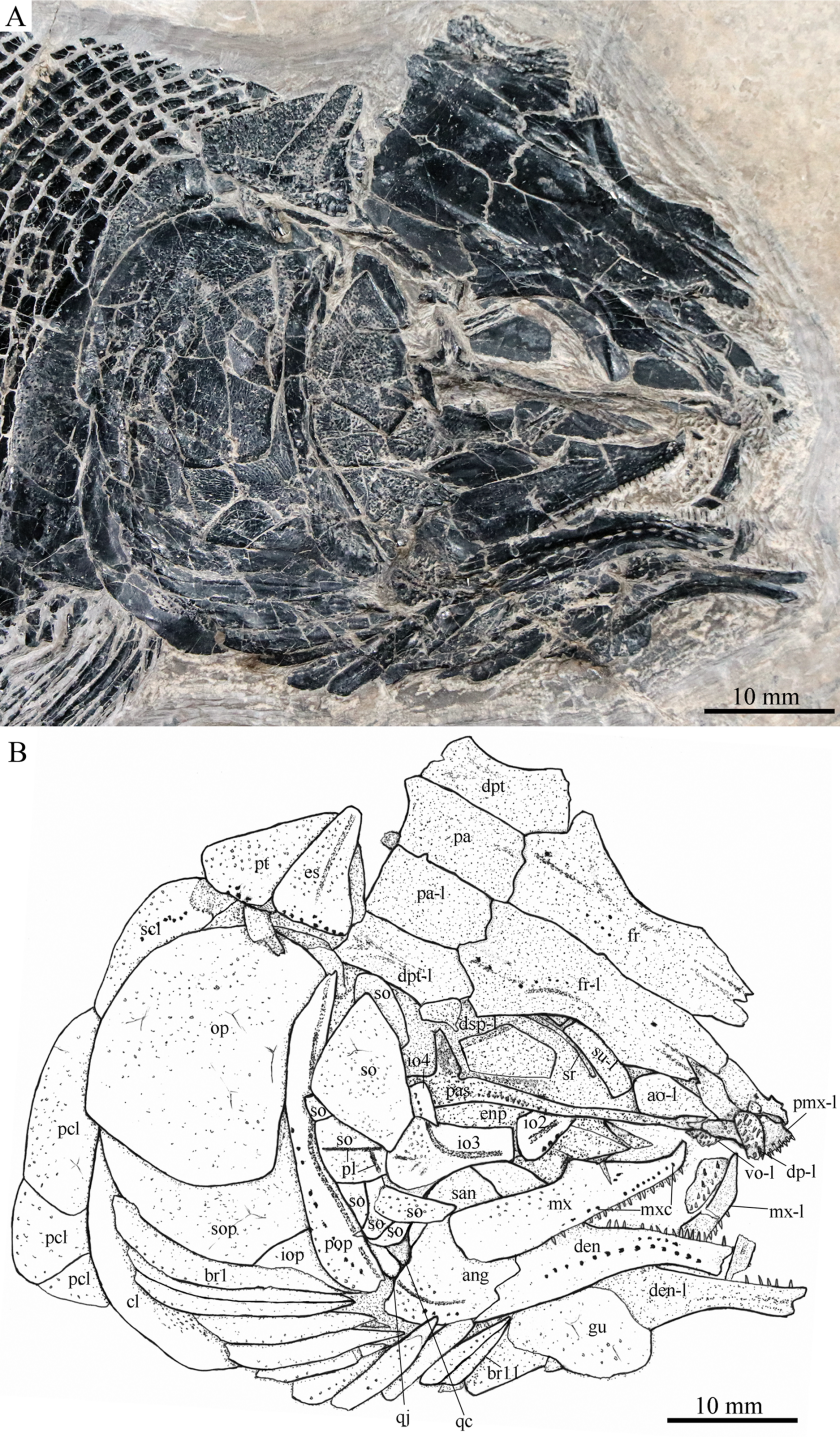
Skull and pectoral girdle in IVPP V20416. Skull and pectoral girdle of *Robustichthys luopingensis*, IVPP V20416. (A) Photograph. (B) Line drawing. Frontals, parietals, dermopterotics and supraorbitals are exposed in ventral view.

**Figure 7:**
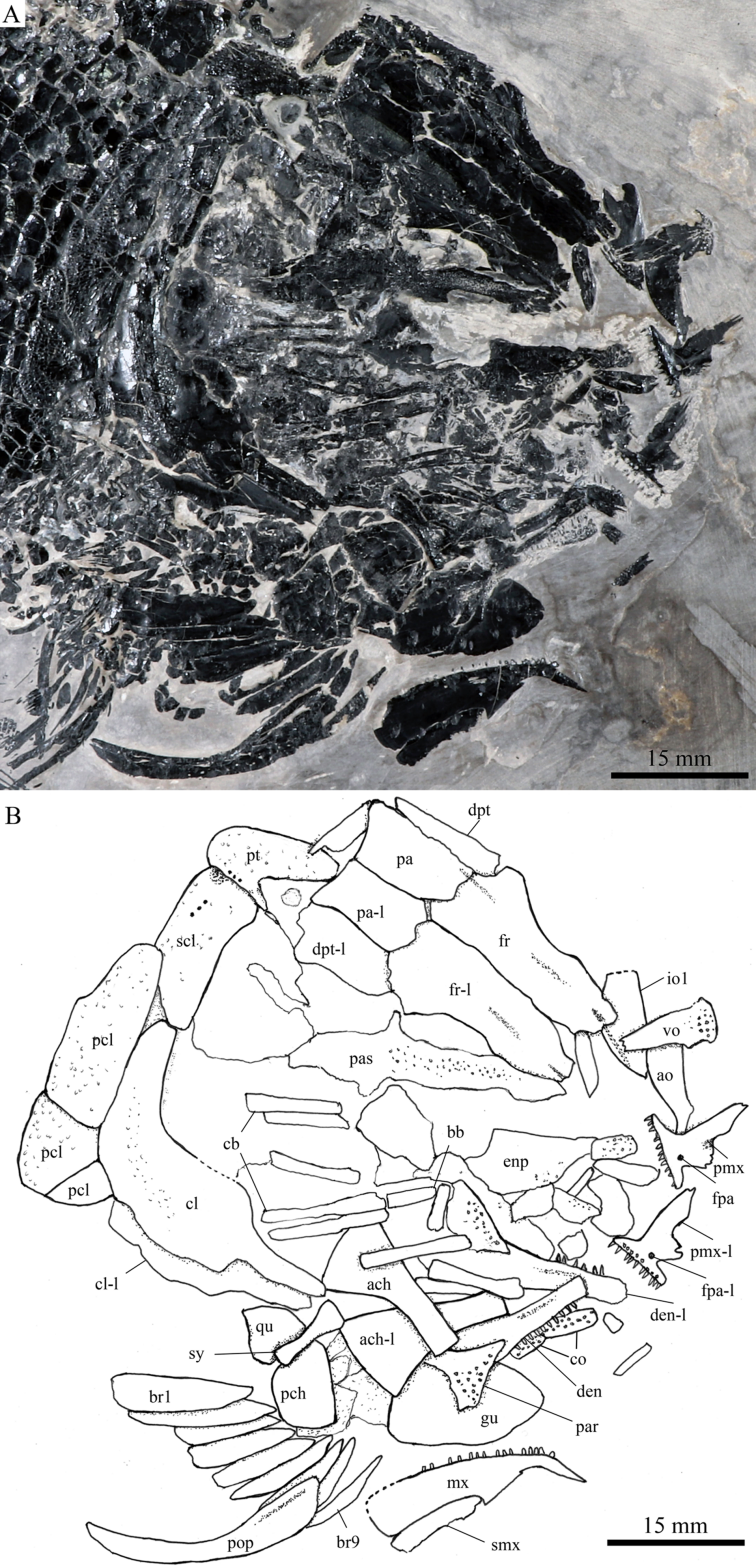
Skull and pectoral girdle in IVPP V18569. Skull and pectoral girdle of *Robustichthys luopingensis*, IVPP V18569. (A) Photograph. (B) Line drawing. Frontals, parietals and dermopterotics are exposed in ventral view.

**Figure 8:**
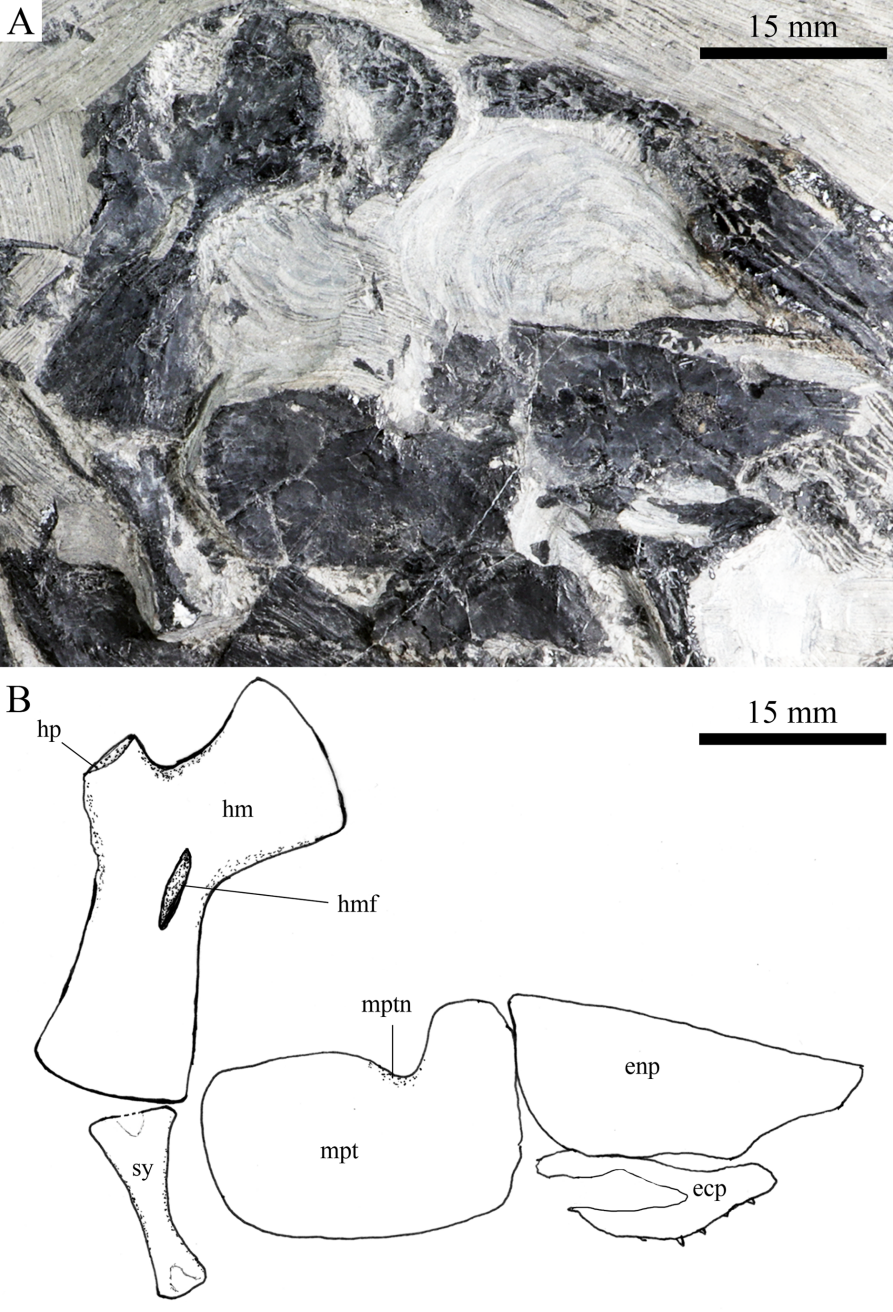
Suspensorium and palatal bones in IVPP V20414. Suspensorium and palatal bones of *Robustichthys luopingensis*, IVPP V20414. (A) Photograph. (B) Line drawing.

**Figure 9:**
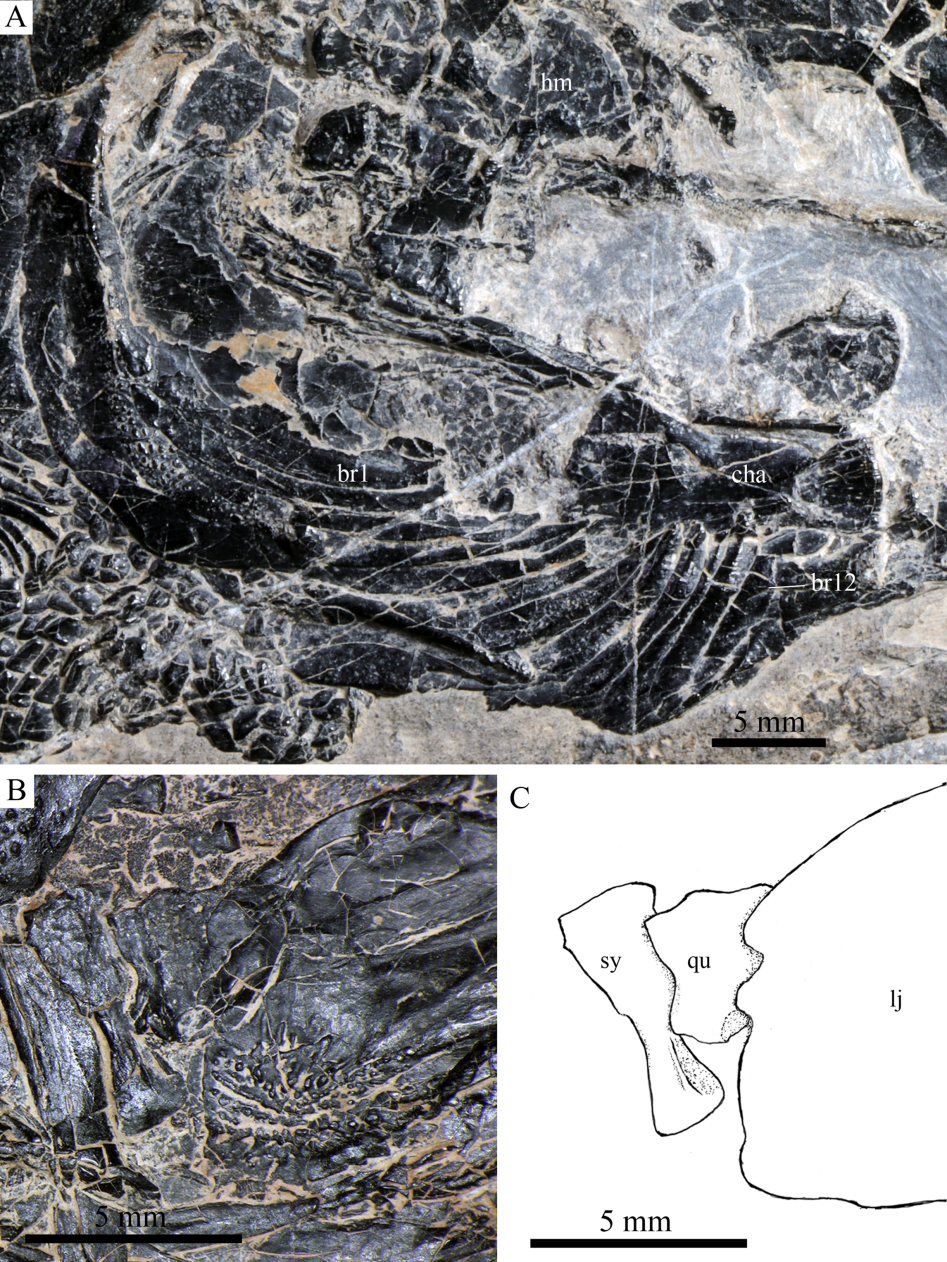
Cranial bones in IVPP V20596 and V18571. Cranial bones of *R. luopingensis*. (A) Hyomandibula, anterior ceratohyals and branchiostegal rays, IVPP V20596. (B and C) Quadrate and symplectic, IVPP V18571. (B) Photograph. (C) Line drawing.

**Figure 10:**
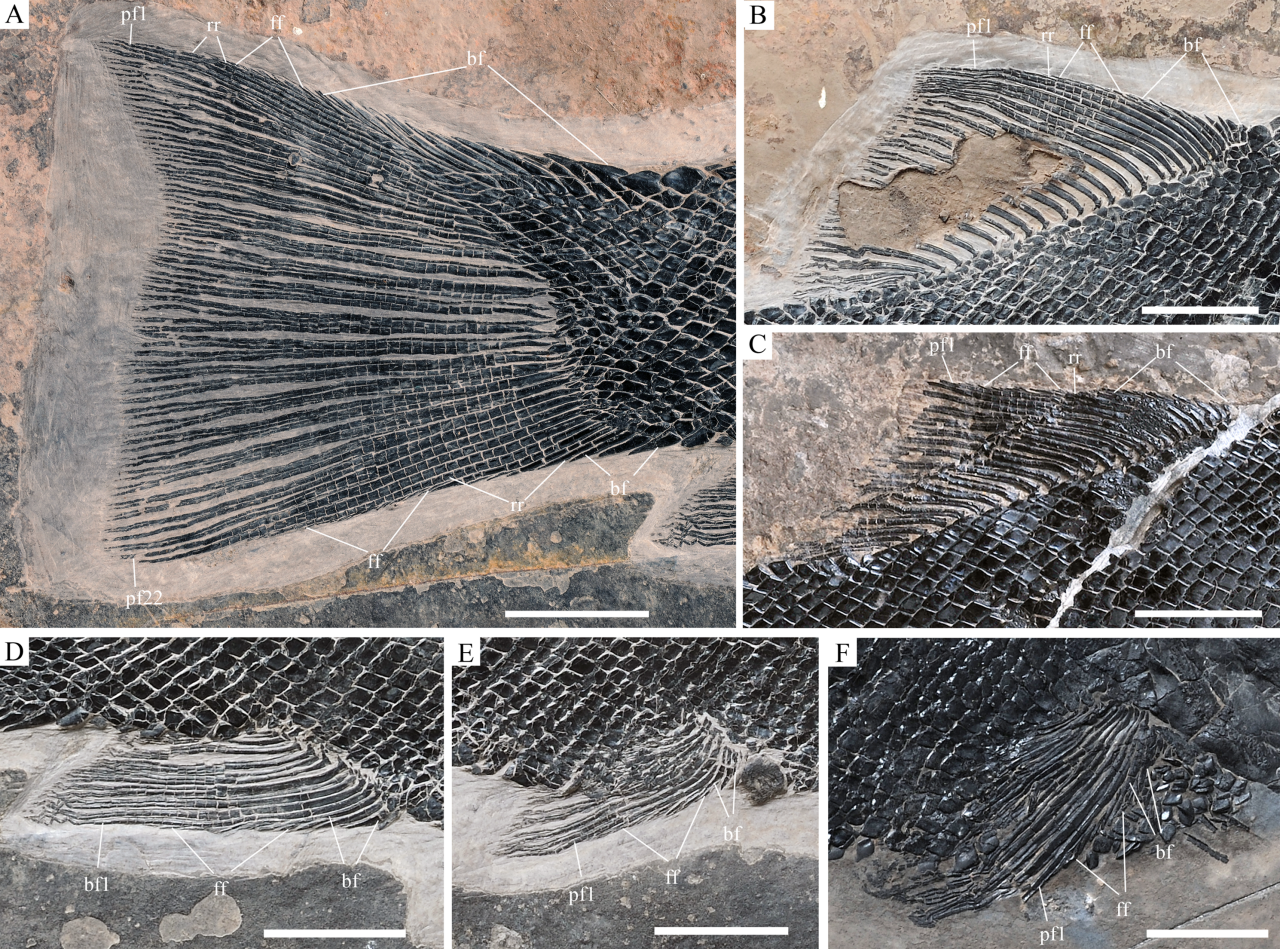
Fins of *Robustichthys*. Fins of *R. luopingensis*. (A) Caudal fin. (B and C) Dorsal fin. (D) Anal fin. (E) Left pelvic fin. (A), (B), (D) and (E) IVPP V18568 (holotype). (C) ZMNH M1691. (F) Left pectoral fin, ZMNH M1690. Scale bars = 10 mm.

**Figure 11:**
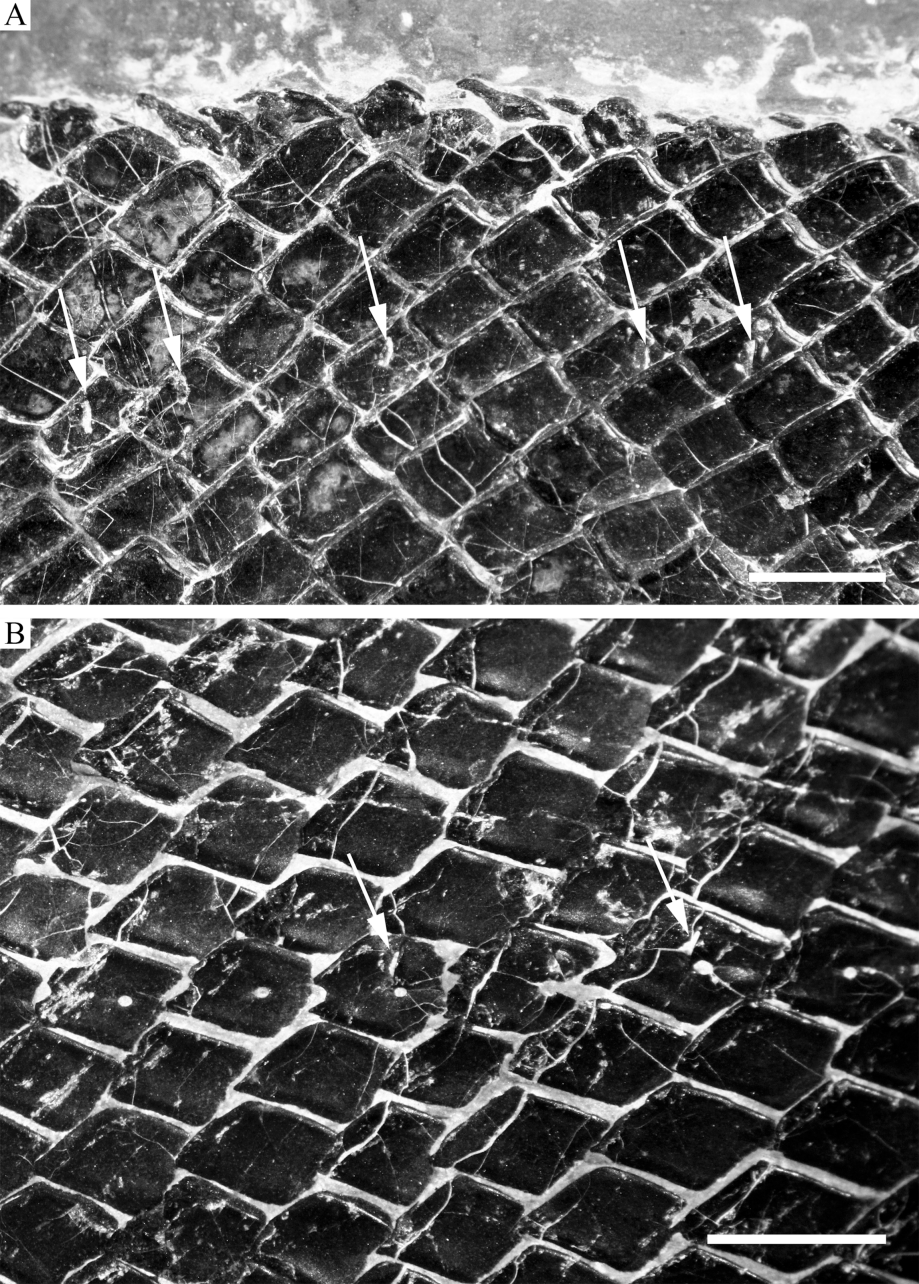
Scales. Scales of *R. luopingensis*. (A) Scales in predorsal region with arrows showing pores of additional lateral line; ZMNH M1691. (B) Main lateral line scales in posterior flank region with arrows showing pit organs, IVPP V18568 (holotype). Immersed in water when photographed. Anterior to right. Scale bars = three mm.

**Holotype**. IVPP 18568, a nearly complete, laterally compressed specimen (Fig. 2A).

**Referred material**. IVPP V18569–18573, 20414–20419, 20594–20596; ZMNH M1690–1691.

**Locality and horizon**. Luoping, Yunnan, China; second (upper) member of Guanling Formation, Pelsonian (∼244 Ma), Anisian, Middle Triassic (Zhang et al., 2009).

**Emended diagnosis**. A large-sized ionoscopiform distinguished from other members of this order by the following combination of features: frontal constricted above orbital region, 2.7 times as long as its maximal width; parietal rectangular, 1.5 times as long as wide; two supraorbitals; dermosphenotic with canal-bearing innerorbital flange; five infraorbitals; seven to nine suborbitals; parasphenoid with relatively small conical teeth; symplectic hourglass-shaped, one-third deeper than quadrate; quadratojugal splint-like, tapering dorsally; maxilla with large supramaxillary process, ending at level of posterior margin of orbit; posterior margin of maxilla nearly straight or very slightly concave; 12 pairs of branchiostegal rays; 11–12 principal rays in each pectoral fin; seven principal rays in each pelvic fin; 19–21 principal dorsal rays; 9–11 principal anal rays; 22–23 principal caudal rays; caudal fin with slightly concave profile; scales rhombic with smooth surface and serrated posterior margin; and scale formula of D26/P12–13, A22–23, C39–40/T48–49.

### Description

#### General morphology and size

*Robustichthys* has a blunt snout, a fusiform body and an abbreviated heterocercal caudal fin with a slightly concaved profile (Fig. 2). The dorsal fin is notably larger than the anal fin, and inserts slightly anterior to the origins of pelvic fins. The holotype (Fig. 2A) is 141 mm in SL (the length from the tip of the snout to the posterior extremity of the caudal peduncle), and the largest specimen (IVPP V20414) reaches a SL up to 360 mm. The head length (measured from the tip of the snout to the posterior margin of the opercle) accounts 27.6–32.1% of SL (Table 1). The greatest body depth lies midway between the posterior margin of the opercle and the origin of the dorsal fin. The outer surfaces of cranial bones except those in the snout region are ornamented with ridges and tubercles.

**Table 1:** Measurement data on nearly complete specimens.

Specimens	SL	HL	BD	PVL	PDL	PAL	TL
V18568	140	45	48	84	81	107	175
V18569	198	55	61	121	117	152	–
V18570	164	54	55	97	95	125	204
V18571	124	41	43	77	73	93	158
V18572	285	80	90	172	170	223	325
V18573	217	70	73	133	126	–	260
V20414	360	100	128	215	210	286	–
V20416	163	53	56	95	90	122	–
V20417	205	67	71	122	118	153	245
V20419	215	65	74	128	122	–	∼260
V20594	158	52	53	92	88	116	195
V20596	157	50	55	95	–	117	–
M1690	168	54	59	100	96	127	204
M1691	155	49	51	94	87	121	192

**Notes:**

Measurement data (in mm).

BD, body depth; HL, head length; PAL, preanal length; PDL, predorsal length; PVL, prepelvic length; SL, standard length; TL, total length.

#### Snout

The canal-bearing bones include a median rostral and a pair of nasals and antorbitals (Figs. 3–6). The median rostral is small and dorsoventrally short, having a sub-circular middle portion and a pair of short lateral processes. The anterior margin is concave and the posterior margin is convex. This bone overlies the anterior portions of the premaxillae, and contacts the nasals posterodorsally and the antorbitals laterally. The anterior commissure of the lateral line system is enclosed in the rostral, indicated by a small pore near the base of each lateral process of this bone.

The nasals are irregular in shape and slightly curved (Figs. 3 and 4). The maximal width is located at the middle-dorsal portion where each nasal reaches the middle line of the skull and contacts its counterpart medially. The anteroventral portion of this bone bends downward and contacts the rostral medially for a short length. The lateral margin of the nasal is notched for the posterior nostril. The anterior nostril is probably located between the nasal, rostral and antorbital, as in the living bowfin. The supraorbital sensory canal in the nasal is indicated by several pores associating with a canal parallel to the lateral margin of this bone (Figs. 3 and 4).

The antorbitals are hook-like, being half of the length of the lower jaw (Fig. 4). Each antorbital has a curved, tube-like anterior arm that transfers the ethmoid commissural canal anteriorly to the rostral, and an expanded posterior portion that contacts the nasal anteriorly, the frontal and supraorbital dorsally, and the first infraorbital ventrally. The infraorbital canal extends posterodorsally and ends at the two-third length of the antorbital. The posterodorsal margin of the antorbital forms part of the orbital margin (Fig. 4).

#### Skull roof

The skull roofing bones include a pair of frontals, parietals, dermopterotic, and extrascapulars (Figs. 3–7). The frontal is elongate and roughly four-sided, with irregular anterior and posterior margins, a nearly straight medial margin and a curved lateral margin (Fig. 6). The bone is constricted above the orbital region, with a triangular lateral expansion near the level of the anterior margin of the orbit. It tapers forward from this expansion and contacts the antorbital laterally and the nasal anteriorly. The length is 2.7 times its maximal width, which lies at the level of the posterior margin of the orbit. The supraorbital sensory canal enters the frontal from the nasal, runs longitudinally through this bone, and enters the parietal posteriorly.

The parietal is nearly rectangular, 1.5 times as long as wide, with narrow transverse zones at its anterior and posterior portions overlapped by the frontal (Figs. 5 and 6) and the extrascapular (Figs. 4 and 5), respectively. In ventral view, the parietal is nearly half of the frontal in length (Figs. 6 and 7). The left parietal contacts the right in a zigzag suture in most specimens (Figs. 3–6). Three pit-lines, best observed in ZMNH M1690 (Fig. 4), are present on each parietal. The anterior pit-line extends anteriorly near the lateral margin of this bone. The middle pit-line originates from the posterolateral portion of the parietal, extends laterally into the dermopterotic, and traverses two thirds of the width of this bone. The posterior pit-line originates above the dorsal tip of the middle pit-line, and extends posteriorly for a short length in this bone.

The dermopterotic is elongate with a nearly straight medial margin and a concave lateral margin (Figs. 3–7). It is 1.2 times as long as the parietal, having a triangular anteromedial process that overlaps the posterior portion of the frontal. The temporal sensory canal runs longitudinally through the dermopterotic near its lateral margin, and posteriorly enters the extrascapular.

The trapezoidal extrascapular tapers medially, with a concave anterior margin and a slightly convex posterior margin (Figs. 3–6). The supratemporal commissure extends transversely through the extrascapular, indicated by several pores associating with a canal at the middle portion of this bone.

#### Circumorbital bones

Two supraorbitals flank the orbital margin of each frontal (Figs. 3–6). Both are elongate and nearly rectangular; the anterior is 1.2–1.4 times as long as the posterior.

Five infraorbitals are present (Figs. 3–6). The first infraorbital (=lachrymal) is cleaver-shaped, twice longer than deep, with a nearly straight anterodorsal margin and a convex ventral margin. The infraorbital sensory canal passes longitudinally through this bone near its ventral margin with a branch running into the maxilla (Figs. 4 and 5).

The second infraorbital is relatively small and trapezoidal, nearly 1.5 times longer than deep. It has a triangular anteroventral portion inserting between the first infraorbital and the maxilla. The infraorbital sensory canal runs longitudinally through this bone at its middle portion. The third infraorbital is large and roughly pentagonal with a rounded posterior margin. The sensory canal extends through this bone at its dorsal portion.

The last two infraorbitals are small. The fourth is trapezoidal, and the fifth is irregular with a posterodorsal projection contacting the dermopterotic (Figs. 3 and 5). The infraorbital sensory canal runs dorsoventrally through both bones near their anterior margins.

The dermosphenotic is trapezoidal, contacting the supraorbtial anteriorly, the dermopterotic and sphenotic posteriorly, and the frontal medially (Figs. 3–5). The lateral margin of the dermosphenotic forms part of the orbital margin. Ventrally, the dermosphenotic bears a short innerorbital flange (IVPP V18571; Xu, Zhao & Coates, 2014: fig. 1e), through which it receives the infraorbital sensory canal from the last infraorbital.

The sphenotic is not fused with the dermosphenotic, having an exposed dermal component on the skull roof (Figs. 3 and 4).

There are eight or nine suborbitals (the number slightly varies in different specimens) between the preopercle and infraorbital bones. The first (uppermost) is elongate with rounded anterior and posterior margins. The second is small and sub-circular, contacting the last infraorbital anteriorly. The third, commonly the largest one, is trapezoidal. The remaining suborbitals vary much in size and shape in different specimens. Two pit-lines, including a horizontal one and a vertical one, are present on these suborbitals (Figs. 3–5).

The sclerotic bones are preserved near the orbital rim (Figs. 3 and 6). They are narrow and curved, and their number cannot be determined because of incomplete preservation.

#### Parasphenoid and vomers

The median parasphenoid, often exposed through the orbit (Figs. 3–7), has a well-developed ascending ramus on each side of its middle-posterior portion. This ramus is dorsoventrally grooved in its lateral margin (Fig. 6), probably for the spiracular canal as in the living bowfin (Grande & Bemis, 1998). The ventral surface of the parasphenoid bears a long tooth patch of dense small conical teeth, which extends from between the ascending rami anterior to the suture with the posterior extent of the vomers. Foramina for the internal carotid or afferent pseudobranchial arteries are absent in this bone.

The paired vomer is elongate and plate-like, bearing a broad tooth patch along the anterior quarter of this bone (Figs. 6 and 7). The teeth are conical, slightly larger than those on the parasphenoid.

#### Palatine, hyoid, and branchial series

The dermopalatines are small and elongate, covered with pointed teeth, which are smaller than those in the maxilla (Fig. 6).

A right metapterygoid, entopterygoid, and ectopterygoid are discernible in lateral view (Fig. 8). The metapterygoid is large and plate-like. The dorsal margin of this bone bears a well-developed notch, through which the trigeminal nerve may pass. The entopterygoid is triangular, tapering anteriorly. Lateral to the entopterygoid is the ectopterygoid, which is relatively small and elongate. Dense small conical teeth are present on the oral margins of these bones (Figs. 7 and 8).

The quadrate is well exposed in IVPP V18569 (Fig. 7) and V18571 (Fig. 9B). It is fan-shaped, articulating with the lower jaw via a strong condyle.

The quadratojugal is small and splint-like, tapering posterodorsally. It rests on the anterior edge of the preopercle, and articulates with the posterolateral surface of the ventral portion of the quadrate (Fig. 3).

A hyomandibula is exposed in IVPP V20414 (Fig. 8) and V20596 (Fig. 9A). It is a large hatchet-shaped bone which articulates with the braincase anterodorsally. The posterior border of the hyomandibula has a knob-like opercular process. A large foramen for the hyomandibular branch of the facial nerve is present near the center of this bone.

The symplectic is well exposed in IVPP V20414 (Fig. 8) and V18571 (Fig. 9B). It is hourglass-shaped, one-third deeper than the quadrate. This bone is not exposed in the holotype; Xu, Zhao & Coates (2014) misidentified the quadratojugal as part of the symplectic in this specimen. Sun et al. (2017), however, misidentified the symplectic in V18571 (Fig. 9B) as a plate-like “quadratojugal.”

A hypohyal is exposed near the anterior ceratohyal in ZMNH M1690 (Fig. 4). It is small and nearly square. As in other holosteans, a foramen for the afferent hyoidean artery is absent in this bone.

The anterior ceratohyal is roughly hourglass-shaped, more expansive posteriorly than anteriorly (Figs. 7 and 9A). The posterior ceratohyal is sub-circular, 40% the length of the anterior ceratohyal, having a rounded posterior margin and a nearly straight anterior margin (Fig. 7).

Elements of the branchial arches are partly exposed, including rod-like hypobranchials and ceratobranchials (Fig. 7), but the poor state of preservation does not permit a precise counting of their number.

#### Jaws

The paired premaxilla is relatively large and deep, having a horizontally expanded oral region and a posterodorsally directed nasal process (Fig. 7). A foramen for the olfactory nerve is absent in the nasal process of this bone, showing a primitive condition as in other early halecomorphs (*Watonulus*, Olsen, 1984; *Asialepidotus*, Xu & Ma, 2018). A small foramen is present slightly above the anterior oral margin of this bone (Fig. 7). According to its position and size, this should be the foramen for the palatine ramus of the facial nerve. A row of 11 teeth are discernible along the oral margin of the premaxilla. Medially, there is an additional row of about 10 teeth. The teeth are conical, with the medial ones slightly smaller than the lateral ones.

The maxilla is elongate, bearing a peg-like, medially-directed anterior process (Figs. 3–7). The anterior-middle portion of this bone protrudes dorsally and forms a triangular supramaxillary process. Posteriorly, the maxilla ends at the level of the posterior margin of the orbit. The posterior margin of the maxilla is nearly straight or very slightly concave, resembling that in *Macrepistius* and *Amblysemius*. As in other ionoscopiforms, the maxilla encloses a branch of the infraorbital sensory canal, indicated by a series of small pores and pits near the oral margin of this bone.

The supramaxilla is elongate, being about half the length of the maxilla (excluding the anterior process).

The lower jaw is elongate and strong, having a height/length ratio of 44%. The wedge-shaped dentary is the largest element of the lower jaw, contacting the supra-angular and angular posteriorly. The supra-angular is small and elongate. The angular is relatively large and trapezoidal with its anterior portion laterally covered by a flange of the dentary. The suture between the angular and dentary is sinuous. Both the supra-angular and dentary contribute the coronoid process. The mandibular canal extends longitudinally through the dentary and angular. A dorsoventrally directed pit line is present on the lateral surface of the posterior portion of the angular.

Medially, at least two coronoid bones and a prearticular are discernible in each lower jaw (Figs. 4 and 7A). The coronoids are small and elongate. The prearticular is large and roughly triangular. The oral margins of the coronoids and prearticular are covered with dense conical teeth. The articular is only partly exposed (Fig. 3), and its complete outline is still unknown. A retroarticular is not discernable; it is unknown if this bone is present.

#### Opercular series

The preopercle is crescent-shaped, with its ventral portion slightly more expanded than the dorsal portion. The dorsal tip of the preopercle nearly contacts the posterolateral process of the dermopterotic (Figs. 3–6). The preopercular sensory canal extends dorsoventrally through the entire length of the preopercle, indicated by a groove that extends near the anterior margin of this bone. In addition, there are some posterior diverticula associating with the sensory canal at the middle-ventral portion of this bone.

The opercle is large and trapezoidal, with rounded posterior, dorsal and ventral margins and a nearly straight anterior margin (Figs. 3–6). It is 1.2–1.3 times deeper than long. A socket-like opercular facet is present on the medial surface of the anterodorsal region of this bone (IVPP V20416). The subopercle is relatively small and sickle-shaped, bearing a triangular anterodorsal process that is slightly less than half of the depth of the opercle. The interopercle is small and triangular, partly overlapped by the preopercle. It tapers anteroventrally, and nearly reaches the posterior end of the lower jaw.

#### Gular and branchiostegal rays

The median gular is sub-circular and slightly tapers anteriorly, being about half of the length of the lower jaw (Figs. 5–7). A transverse pit-line and several pores are present on the external surface of this bone (Fig. 5).

A total of 10 and 11 right branchiostegal rays are preserved in the holotype (Fig. 3) and IVPP V20416 (Fig. 6), respectively. In addition, 12 right branchiostegal rays are preserved in V18570 and V20596 (Fig. 9A), representing the maximum number in this taxon. They are elongate and plate-like, increasing in length and width posteriorly.

#### Girdles and paired fins

The posttemporal is sub-triangular with a rounded posterolateral margin (Figs. 3–7). It tapers medially, having a narrow anterior region overlapped by the extrascapular. Ventrally, the posttemporal bears a long, anteriorly directed process that probably contacts the intercalar (Figs. 6 and 7). The lateral line penetrates the anterior half of this bone near its lateral margin, extends ventrally, and enters the supracleithrum.

The supracleithrum is deep and anteriorly inclined. The dorsal portion of this bone has a distinct, concave articular facet for articulation with the posttemporal (Figs. 6 and 7). The presupracleithrum is small and sub-circular (Fig. 3). The cleithrum is large and sickle-shaped, with the horizontal branch slightly shorter than the vertical one. There are three postcleithra. The dorsal is large and rhombic, nearly as deep as the supracleithrum; the middle is trapezoidal, slightly less than half of the depth of the dorsal; and the ventral is small and triangular.

The pectoral fins insert low on the body, and each bears 11–12 distally segmented rays (Fig. 10F). The first is unbranched, preceded by two or three basal fulcra. The remaining rays are branched distally.

The pelvic girdles are not exposed. The pelvic fins insert at the 13th vertical scale row. Each bears seven distally segmented rays, preceded by three or four basal fulcra (Fig. 10E).

Small, elongate fringing fulcra are present on all paired fins.

#### Median fins

The dorsal fin originates above the 26th vertical scale row. It is composed of 19–21 principal rays (Figs. 10B and 10C). The first ray is unbranched, and the remaining rays are branched distally. A rudimentary ray is commonly present, and its length varies in different specimens; it is three-fourths the length of the first principal ray in the holotype (Fig. 10B) and is half the length of the latter in ZMNH M1691 (Fig. 10C). Anterior to the rudimentary ray are seven or eight basal fulcra.

The anal fin originates below the 23rd vertical scale row and is composed of 9–11 principal rays (Fig. 10D). The first is unbranched, preceded by a rudimentary ray and two to four basal fulcra; and the remaining rays are branched distally. The rudimentary ray consists of only three segments, being about one-third the length of the first principal ray.

The caudal fin is abbreviated heterocercal with a slightly forked profile (Fig. 10A). It is composed of 12–14 basal fulcra, two rudimentary rays, and 11 principal rays in the dorsal lobe, and three or four basal fulcra, two to five rudimentary rays, and 11 or 12 principal rays in the ventral lobe. The marginal principal rays are unbranched and the middle ones are branched up to three times. The articulations between the segments of all rays are straight. Fringing fulcra are present in all fins.

#### Squamation

The body is fully covered with rhomboid scales (Fig. 2). The scales are arranged in 43–45 vertical rows along the main lateral line. In addition, there are about ten inverted rows of scales posterior to the hinge line in the caudal region. The 23rd vertical row (above which the dorsal fin originates) is composed of 29–30 scales (15 above the lateral line). The anterior scales in the middle flank region are 1.2 times deeper than wide, and they gradually become shorter and smaller dorsally, ventrally, and posteriorly. The scales are largely smooth; most of them have a slightly serrated posterior margin with two to five small serrations, but those in the caudal region have a straight posterior margin. A dorsal peg and anterodorsal extension are present on anterior flank scales. Each of the main lateral line scales has a small sensory pore near its central part. Moreover, every two to four lateral line scales has an additional, dorsoventrally elongate pore at its dorsal portion (Fig. 11B). These pores probably represent individual pit organs that are separate and independent from the lateral line canal (Schultze, 1966). A similar condition is present in the ionoscopiform *Ophiopsiella* (Lane & Ebert, 2015). Besides the main lateral line, an additional lateral line is present in the predorsal region, indicated by a line of seven or eight small pores on the scales in this region (Fig. 11A).

## Discussion

### Revised reconstruction and comparison

**Figure 12:**
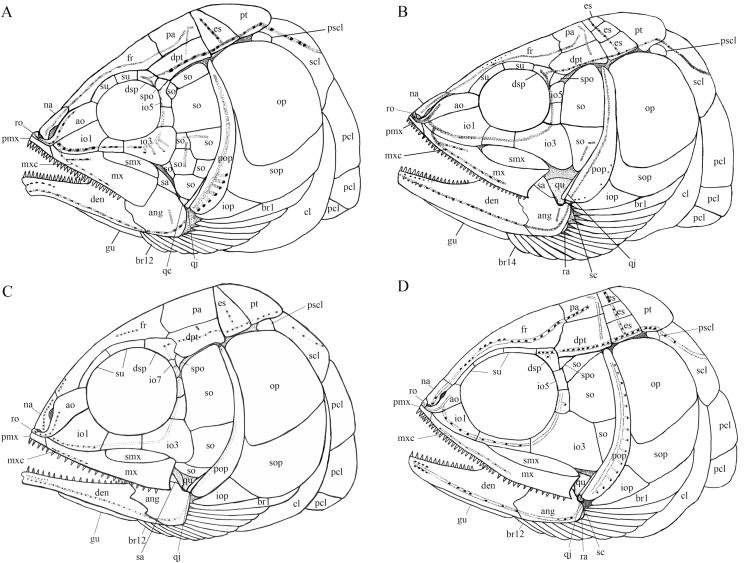
Comparison of skull and pectoral girdle of four ionoscopiforms from the Middle Triassic of China. Comparison of skull and pectoral girdle of Middle Triassic ionoscopiforms. (A) *Robustichthys*. (B) *Asialepidotus*. (C) *Panxianichthys*. (D) *Subortichthys*. Figures not drawn to the same scale.

**Figure 13:**
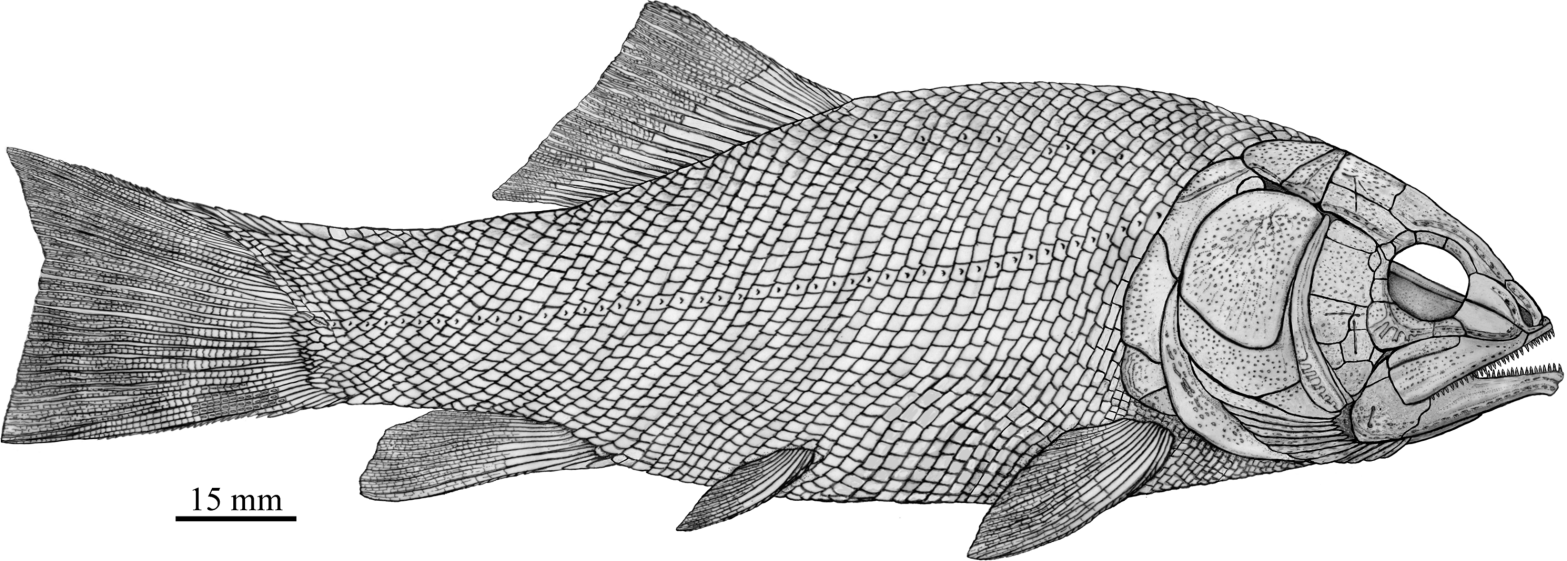
Reconstruction of *R. luopingensis*.

A comparative study of new material with type specimens has revealed many previously unknown or incompletely known anatomical details on the skull of *Robustichthys* (e.g., sclerotic bones, hyomandibula, symplectic, quadratojugal, anterior and posterior ceratohyals, parasphenoid, vomer, palatal bones, premaxilla, prearticular, and complete series of branchiostegal rays). The new data have permitted a revision of the cranial anatomy (Fig. 12) and life reconstruction (Fig. 13) of *Robustichthys*. This revision is significant in reassessing the phylogenetic position of *Robustichthys* within the Holostei. For example, López-Arbarello, Stockar & Bürgin (2014) placed *Robustichthys* in the “Furidae” (=Ophiopsidae, Bartram, 1975; Lane & Ebert, 2012, 2015) mainly based on the “absence” of sclerotic bones in this taxon. My revision, however, shows that the sclerotic bones are actually present in *Robustichthys* (Figs. 3 and 6), resembling those in *Panxianichthys* (Xu & Shen, 2015) and *Asialepidotus* (Xu & Ma, 2018). Sun et al. (2017) argued that *Robustichthys* was affiliated with ginglymodians in lacking observable double joint and palatal dentation. However, my revision shows that *Robustichthys* do have an *Amia*-like jaw joint and palatal dentation. Ebert (2018) followed Sun et al.’s (2017) identification of the symplectic in IVPP V18571 as a plate-like “quadratojugal” and placed *Robustichthys* in the paraphyletic “Panxianichthyiformes.” Indeed, both *Robustichthys* and *Panxianichthys* have a splint-like quadratojugal, resembling that in *Subortichthys* (Ma & Xu, 2017) and *Asialepidotus* (Xu & Ma, 2018).

A plate-like quadratojugal was previously identified by Olsen (1984) in *Watsonulus*, but this was not confirmed by Gardiner, Maisey & Littlewood (1996), who found no trace of a rectangular quadratojugal as figured by Olsen (1984) in any parasemionotid specimens examined by them from Madagascar and eastern Greenland. Instead, Gardiner, Maisey & Littlewood (1996) confirmed Patterson’s (1973) observations on parasemionotids that there is a vertical flange of membrane bone projecting fore and aft on the outer surface of the quadrate, which likely represents a reduced quadratojugal fused to the quadrate; the same authors further concluded that this kind of quadratojugal is also present in non-amiid halecomorph *Caturus*, *Oshunia* and *Ionoscopus* (Bartram, 1977; Lambers, 1992). In addition, Bartram (1977) and Lombardo (2001) described a splint-like quadratojugal in *Furo longiserratus* and *Allolepidotus* within halecomorphs, respectively. It is now evident that a splint-like quadratojugal is present in all four ionoscopiform halecomorphs from the Middle Triassic of China (Fig. 12). Outside of the halecomorph clade, a splint-like quadratojugal is otherwise present in ginglymodians. The identification of a splint-like quadratojugal in early halecomorphs indicates that this feature is not uniquely derived for ginglymodians as previously suggested (Grande, 2010; López-Arbarello & Sferco, 2018). A quadratojugal is independently lost in amiid halecomorphs and teleosts. The posterodorsal process of the quadrate in teleosts was once interpreted as a quadratojugal fused to the quadrate (Allis, 1909), but this lacks support from ontogenetic evidence (Arratia & Schultze, 1991).

Patterson (1973) first proposed the double jaw joint (involving both symplectic and quadrate) as a halecomorph synapomorphy, and his hypothesis is widely accepted by many authors (Gardiner, Maisey & Littlewood, 1996; Grande & Bemis, 1998; Alvarado-Ortega & Espinosa-Arrubarrena, 2008; Lane & Ebert, 2012; Brito & Alvarado-Ortega, 2013; Xu, Zhao & Coates, 2014; Xu, Ma & Ren, 2018a; López-Arbarello, Stockar & Bürgin, 2014; Xu & Shen, 2015; Taverne, 2015; Ma & Xu, 2017). Olsen (1984) noticed Nielsen’s (1942, 1949) descriptions of the symplectic articulating with the lower jaw in non-neopterygian *Birgeria*, *Boreosomus* and *Pteronisculus*, and challenged Patterson’s (1973) hypothesis in suggesting that the double jaw joint is plesiomorphic for halecomorphs. However, the identification of the symplectic is doubtful in non-neopterygians. We follow Gardiner, Maisey & Littlewood (1996) reinterpretations that the previously alleged “symplectic” in *Birgeria*, *Boreosomus* and *Pteronisculus* (Nielsen, 1942, 1949) is an interhyal and the “interhyal” a posterior ceratohyal. Outsides of halecomorphs, the articulation of symplectic with the lower jaw is known from the aspidorhynchid *Vinctifer* (Brito, 1988; but see Maisey, 1991) and pycnodonts (Nursall & Maisey, 1991), but this likely represents convergent evolution (Gardiner, Maisey & Littlewood, 1996; Brito & Alvarado-Ortega, 2013). Recently, Arratia (2013) described that the symplectic also articulates with the lower jaw in the pholidophorid *Pholidophorus gervasutti*, but this condition, unknown in other early teleosts, probably represents another convergent evolution. The double jaw joint has not been known in any ginglymodians. In addition, the symplectic is generally splint-shaped or rode-like in early ginglymodians and teleosts, and is L-shaped in living gars (Grande, 2010). In comparison, the symplectic in halecomorphs is hourglass-shaped or hatchet-shaped. The presence of an hourglass-shaped symplectic articulating with the lower jaw strongly supports the classification of the holostean *Robustichthys* within the Halecomorphi.

### Phylogenetic position of *Robustichthys* within Halecomorphi

**Figure 14:**
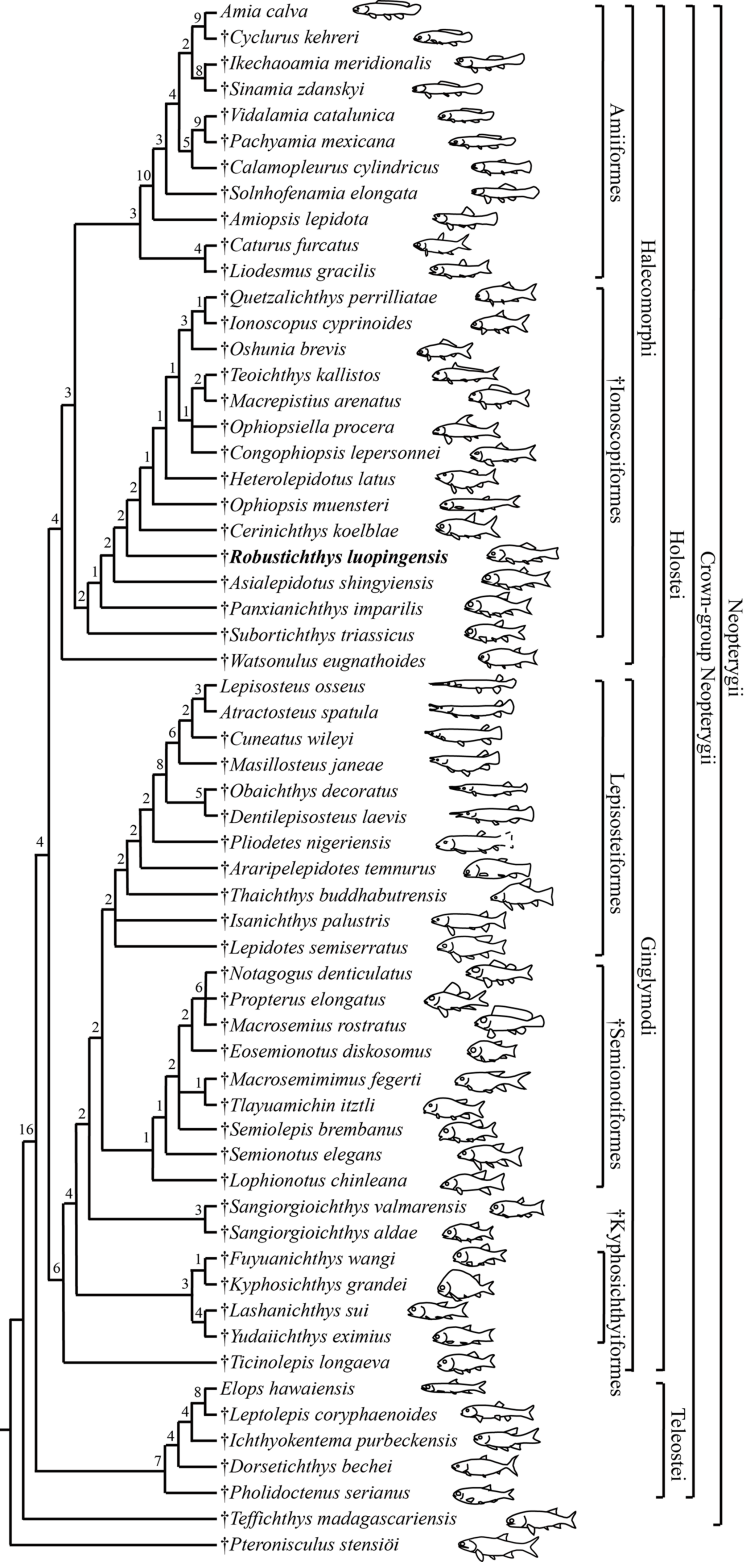
Strict consensus of 24 most parsimonious trees. Strict consensus of 24 most parsimonious trees (tree length = 650 steps, consistency index = 0.4185, retention index = 0.7680), illustrating the phylogenetic relationships of *Robustichthys* within the Neopterygii. Digits above nodes indicate Bremer decay indices. For character descriptions and data matrix, see the online Supplemental Information.

My analysis resulted in 24 most parsimonious trees (tree length = 650 steps, consistency index = 0.4185, retention index = 0.7680), a strict consensus of which is presented in Fig. 14. Four halecomorphs from the Middle Triassic of South China, *Subortichthys*, *Panxianichthys*, *Asialepidotus* and *Robustichthys* are recovered successively at the base of the monophyletic Ionoscopiformes (consistent with Xu & Ma (2018) and Xu et al. (2019)); consequently, the monophyly of Sun et al.’s (2017) “Panxianichthiformes” is not supported. *Robustichthys* possesses several derived features of ionoscopiforms: presence of a lateral line canal in the maxilla, presence of a splint-like quadratojugal (independently evolved in ginglymodians; secondarily lost in *Cerinichthys* and more derived ionoscopiforms), presence of a relatively long parietal (absent in *Subortichthys*), presence of a canal-bearing innerorbital flange of the dermosphenotic (absent in *Subortichthys* and *Panxianichthys*), presence of a sphenotic with a relatively large exposed dermal component nearly reaching the orbital margin (absent in *Subortichthys*), orbital length no longer than the pre-orbital length (absent in *Subortichthys* and *Panxianichthys*; independently evolved in ginglymodians), and presence of a posteriorly inclined last infraorbital (absent in *Subortichthys*, *Panxianichthys*, and *Asialepidotus*). However, it lacks several derived features of *Cerinichthys* and remaining ionoscopiforms: presence of three or more supraorbitals (independently evolved in *Subortichthys*; lost in *Oshunia*), presence of a dermosphenotic firmly sutured into skull roof (independently evolved in amiiforms), absence of a splint-like quadratojugal (independently evolved in ginglymodians), and absence of a supramaxilla process of maxilla (independently evolved in some amiiforms and derived ginglymodians).

Results of two previous analyses (López-Arbarello, Stockar & Bürgin, 2014; López-Arbarello & Sferco, 2018) recovered *Robustichthys* as a sister taxon to *Archaeosemionotus* within the Halecomorphi. This sister taxon relationship is supported in the first analysis (López-Arbarello, Stockar & Bürgin, 2014) by presence of strongly ornamented dermal skull bones and absence of a postmaxillary notch, and in the second (López-Arbarello & Sferco, 2018) by absence of a quadratojugal. However, we do not think the ornamentation of skull bones means much because it is a primitive feature widely present in early holosteans (Olsen, 1984; Tintori et al., 2010; López-Arbarello et al., 2011; Xu & Wu, 2012; Wen et al., 2012; Xu & Ma, 2018). As for the postmaxillary notch, it is a little complex. A postmaxillary notch is primitively absent in neopterygians. Presence of this notch has been regarded as a halecomorph synapomorphy (Grande & Bemis, 1998), but it is secondarily lost in several halecomorphs (e.g., *Amblysemius*, Grande & Bemis, 1998; *Cipactlichthys*, Brito & Alvarado-Ortega, 2013). It is hard to know if the absence of the postmaxillary notch in *Robustichthys* and *Archaeosemionotus* is homologous. As for the quadratojugal, my reexaminations show that the splint-like quadratojugal is actually present in *Robustichthys*. Hence, the sister taxon relationship between *Robustichthys* and *Archaeosemionotus* currently lacks supportive evidences. Many phylogenetically important cranial features (e.g., rostral, nasal, antorbital, parietal, extrascapular, symplectic, quadratojugal, and sensory canal in maxilla) are unknown in *Archaeosemionotus*, because of incomplete preservation (López-Arbarello, Stockar & Bürgin, 2014). *Archaeosemionotus* remains a halecomorph or holostean incertae sedis (Sun et al., 2017; Ebert, 2018) and urgently needs a further revision. I did not included this problematic taxon in the current analysis, following recent others (Sun et al., 2017; Ma & Xu, 2017; Sun & Ni, 2018; Ebert, 2018).

Results of my analysis show that *Robustichthys* is phylogenetically distant from Ginglymodi, because it possesses many derived features of ionoscopiform halecomorphs mentioned above but lacks ginglymodian synapomorphies, for example, presence of anterior infraorbitals, presence of six or more infraorbitals between the antorbital and the dermosphenotic, and presence of no more than nine pairs of branchiostegal rays. The three characters listed by Sun & Ni (2018) supporting the sister group relationships of *Robustichthys* with other ginglymodians are either the one miscoded in *Robustichthys* (nasals very narrow, separated medially) or those widely distributed in neopterygians (presence of a well-developed posteroventral process of the dentary, and series of denticles along the ridge between the branchial and lateral surfaces of the cleithrum). Additionally, Sun et al. (2017) noticed that *Robustichthys* resembles some ginglymodians in having mosaic suborbitals, but my analysis shows that this feature is independently evolved in ginglymodians and some ionoscopiforms (Applegate, 1988; Machado et al., 2013). As such, the previous placement of *Robustichthys* as a basal ginglymodian (Sun et al., 2017; Sun & Ni, 2018) is not supported.

### Reassessment of amiiform phylogeny and implications

Results of my analysis support the sister group relationship between the Caturidae (represented by *Caturus* and *Liodesmus*) and Amiidae (consistent with Grande & Bemis, 1998; Xu, Zhao & Coates, 2014; Xu & Ma, 2018; Ebert, 2018; but see López-Arbarello & Sferco, 2018). This relationship (or the amiiform monophyly) is supported by several derived characters shared by both clades, for example, absence of an opisthotic, absence of a pterotic, presence of a foramen for the olfactory nerve in the premaxilla (independently evolved in derived ginglymodians; secondarily lost in Macrosemiidae), and presence of a cleithrum with the anterior arm longer than the dorsal arm. Ebert (2018) recovered the Ionoscopidae as the sister group of the Caturidae-Amiidae clade based on a single feature, presence of “amioid-type” scales. However, this feature independently occurs in different lineages of sarcopterygians and actinopterygians (Schultze, 2015). The Ionoscopidae lack the derived features of the Amiiformes (Grande & Bemis, 1998; Xu & Ma, 2018). A wealth of derived features mentioned above supports that the Ionoscopidae are more closely to the Ophiopsidae than to the Amiiformes. Consequently, the previous placement of Ionoscopidae at the base of the revised Amiiformes (Ebert, 2018) is not supported.

Within the Amiidae, the sister group relationship between Sinamiinae (*Sinamia* and *Ikechaoamia*) and Amiinae (*Cyclurus* and *Amia*) is newly recognized here, and this relationship is supported by two derived features: presence of a frontal contributing to the orbital margin (independently evolved in *Oshunia* within halecomorphs), and absence of sclerotic ring ossifications (independently evolved in derived teleosts and ginglymodians). This revised topology provides new insights into the historical paleoecology of halecomorph fishes. Fossil evidence of early halecomorphs, including parasemionotiforms, ionoscopiforms and caturid amiiforms, are known exclusively in marine deposits, indicating that the clade Halecomorphi was originally a marine fish group. Within the Amiidae, Solnhofenamiinae are known exclusively in marine deposits; Amiopsinae and Vidalamiinae are largely marine fishes with a few forms known from freshwater deposits near a marine coastal region; and Sinamiinae and Amiinae represent two exceptions that only lived in fresh water (Grande & Bemis, 1998). Based on the traditional hypothesis of Grande & Bemis (1998), the Sinamiinae were considered phylogenetically distant from the Amiinae; both clades independently adapted to freshwater environments. However, if my new hypothesis is accepted, it appears that the common ancestor of Amiinae and Sinamiinae invaded freshwater environments once and adapted to the freshwater environments before it diverged into two clades.

## Conclusions

Comparative studies of the original fossil material with nine new specimens of *R. luopingensis* have revealed a lot of new and detailed anatomical information, for example, vomers, parasphenoid, premaxillae, sclerotic bones, palatine bones, quadratojugal, hyomandibula, symplectic, anterior and posterior ceratohyals, and branchiostegal rays. The new data have permitted a revision of the cranial anatomy and life reconstruction of *Robustichthys*. The results of a phylogenetic analysis incorporating these new anatomical data confirmed the recovery of *Robustichthys* as a basal ionoscopiform within the Halecomorphi. The previous placements of *Robustichthys* as a basal ginglymodian and the Ionoscopidae as a basal amiiform clade are rejected. The sister group relationship between Caturidae and Amiidae is supported. Within the Amiidae, however, the Sinamiinae is recovered as the sister clade to the Amiinae rather than as the basal clade of this family as previously suggested. The revised topology provides new insights into the evolution and historical paleoecology of halecomorph fishes.

## Supplemental Information

10.7717/peerj.7184/supp-1 Supplemental Information 1 224 morphological characters coded across 60 taxa. Raw data of character-taxon matrix in NEXUS format.

10.7717/peerj.7184/supp-2 Supplemental Information 2 Supplementary data used in the phylogenetic analysis. Material examined and references, characters and character states, dataset and strict consensus.

## Anatomical Abbreviations

ang: angular
art: articular
an: anterior nostril
ao: antorbital
apl: anterior pit line
bf: basal fulcrum
br: branchiostegal ray
cb: ceratobranchial
cha: anterior ceratohyal
chp: posterior ceratohyal
cl: cleithrum
co: coronoid
den: dentary
dp: dermopalatine
dpt: dermopterotic
dsp: dermosphenotic
ecp: ectopterygoid
enp: entopterygoid
es: extrascapular
ff: fringing fulcrum
fpa: fenestra for the palatine ramus of the facial nerve in premaxilla
fr: frontal
gu: gular
hb: hypobranchial
hh: hypohyal
hm: hyomandibula
hmf: foramen and groove for the hyomandibular trunk
hp: opercular process of hyomandibula
io: infraorbital
iop: interopercle
lj: lower jaw
mpl: middle pit line
mpt: metapterygoid
mx: maxilla
mxc: sensory canal in maxilla
na: nasal
op: opercle
pa: parietal
par: prearticular
pas: parasphenoid
pcl: postcleithrum
pn: posterior nostril
pl: pit line
ppl: posterior pit line
pr: principle fin ray
pscl: presupracleithrum
pmp: posterior mandibular pit line
pmx: premaxilla
pop: preopercle
pt: posttemporal
qc: quadrate condyle
qu: quadrate
ra: retroarticular
ro: rostral
rr: rudimentary fin ray
sa: supra-angular
scl: supracleithrum
sr: sclerotic bone
smx: supramaxilla
so: suborbital
sop: subopercle
spo: sphenotic
su: supraorbital
vo: vomer.

## Additional Information and Declarations

### Data Availability

The following information was supplied regarding data availability: The raw data are available in the Supplemental Files. All but two specimens described in this paper are curated at the fossil collections of the Institute of Vertebrate Paleontology and Paleoanthropology (IVPP V18568–18573, 20414–20419, 20594–20596), Chinese Academy of Sciences. The remaining two specimens are at the Zhejiang Museum of Natural History (ZMNH M1690–1691), Hangzhou, China.
