# Supplementary material for: Osteology and phylogeny of Robustichthys luopingensis, the largest holostean fish in the Middle Triassic
Source: PeerJ. 2019 Jun 24;7:e7184. doi: 10.7717/peerj.7184 (PMC6596411; doi:10.7717/peerj.7184)
Supplement: Supplemental Information 2 — Material examined and references, characters and character states, dataset and strict consensus. [file peerj-07-7184-s002.docx]

SUPPLEMENTARY DATA

**Osteology and phylogeny of *Robustichthys luopingensis*, the largest holostean fish in the Middle Triassic**

Guang-Hui Xu^1,2^

^1^Key Laboratory of Vertebrate Evolution and Human Origins of Chinese Academy of Sciences, Institute of Vertebrate Paleontology and Paleoanthropology, Chinese Academy of Sciences, Beijing 100044, China

^2^CAS Center for Excellence in Life and Paleoenvironment, Beijing, 100044, China

Part A Material examined and references

*Amia calva* and *Solnhofenamia elongata* (Grande and Bemis, 1998);

*Araripelepidotes temnurus* (Maisey, 1991; Thies, 1996);

*Asialepidotus shingyiensis* (Xu and Ma, 2018);

*Atractosteus spatula*, *Cuneatus wileyi*, *Dentilepisosteus laevis*, *Lepisosteus osseus*, *Masillosteus janeae*, and *Obaichthys decoratus* (Grande, 2010);

*Caturus furcatus* (Patterson, 1975; Lambers, 1992; Grande and Bemis, 1998; FMNH UC2057);

*Cerinichthys koelblae* (Ebert, 2018);

*Congophiopsis lepersonnei* (Taverne, 2015);

*Dorsetichthys* (‘*Pholidophorus*’) *bechei* (Patterson, 1975; Grande and Bemis, 1998; Arratia, 2013);

*Elops hawaiensis* (Forey, 1973);

*Eosemionotus diskosomus* (López-Arbarello et al., 2019);

*Fuyuanichthys* *wangi* (Xu et al., 2018);

*Heterolepidotus latus* (Brough, 1939; Gardiner, 1960);

*Ichthyokentema purbeckensis* (Griffith and Patterson, 1963);

*Ionoscopus cyprinoides* (Grande and Bemis, 1998; Maisey, 1999; FMNH P15472);

*Isanichthys palustris* (Cavin and Suteethorn, 2006);

*Kyphosichthys* *grandei* (Xu and Wu, 2012; Sun and Ni, 2018);

*Lashanichthys* (‘*Sangiorgioichthys*’) *sui* (López-Arbarello et al., 2011);

*Lepidotes semiserratus* (Stensiö, 1932; Rayner, 1948; Patterson, 1975);

*Leptolepis coryphaenoides* (Patterson, 1975);

*Lophionotus chinleana* (Gibson, 2013)

*Macrepistius arenatus* (Schaeffer 1960, 1971; AMNH 2435)

*Macrosemius rostratus*, *Notagogus denticulatus* and *Propterus elongatus* (Bartram, 1977);

*Macrosemimimus fegerti* (Schröder et al., 2012);

*Ophiopsiella* (‘*Ophiopsis*’) *procera* (Bartram, 1975; Lane and Ebert, 2015; FMNH UC2037);

*Ophiopsis muensteri* (Lane and Ebert, 2012);

*Oshunia brevis* (Maisey, 1991, 1999; AMNH 12000, 12793)

*Panxianichthys imparilis* (Xu and Shen, 2015);

*Pholidoctenus serianus* (Arratia, 2013);

*Pliodetes nigeriensis* (Wenz, 1999);

*Pteronisculus stensiöi* (Nielsen, 1942);

*Quetzalichthys perrilliatae* (Alvarado-Ortega and Espinosa-Arrubarrena, 2008);

*Robustichthys luopingensis* (Xu et al., 2014b; IVPP V18568–18573; 20414–20419, 20594–20596; ZMNH M1690, M1691)

*Sangiorgioichthys aldae* (Tintori and Lombardo, 2007);

*Sangiorgioichthys valmarensis* (Lombardo et al., 2012)

*Semiolepis brembanus* (Lombardo and Tintori, 2008);

*Semionotus elegans* (Olsen and McCune, 1991);

*Subortichthys triassicus* (Ma and Xu, 2017);

*Teffichthys* (‘*Perleidus*’) *madagascariensis* (Lehman, 1952; Marramà et al., 2017; NHMUK P16247, 16248, 19580–19584, 19587–19592, 19595–19599, 19603–19620, 19622, 19623);

*Teoichthys kallistos* (Applegate, 1988)

*Thaiichthys buddhabutrensis* (Cavin et al., 2013);

*Ticinolepis longaeva* (López-Arbarello et al., 2016);

*Tlayuamichin itztli* (López-Arbarello and Alvarado-Ortega, 2011);

*Watsonulus eugnathoides* (Olsen, 1984; Grande and Bemis, 1998);

*Yudaiichthys eximius* (Xu et al., 2019).

Institutional abbreviations: AMNH, American Museum of Natural History, New York, USA; NHMUK, Natural History Museum, London, UK; FMNH, Field Museum of Natural History, Chicago, USA; IVPP, Institute of Vertebrate Paleontology and Paleoanthropology, Chinese Academy of Sciences, Beijing, China; ZMNH, Zhejiang Museum of Natural History, Hangzhou, China.

Part B Characters and character states

**Neurocranium**

1. Post-temporal fossa

(Grande, 2010, Xu and Gao, 2011; Xu and Wu, 2012; Xu et al., 2012, 2014a,b, 2015, 2018, 2019; López-Arbarello, 2012; Cavin et al., 2013; Deesri et al., 2014, 2016; Giles et al., 2017)

- - 1. Absent
    2. Present

1. Vagal foramen

(Modified from Gardiner et al., 1996; Cavin and Suteethorn, 2006; Hurley et al., 2007; López-Arbarello, 2012; Giles et al., 2017; López-Arbarello and Sferco, 2018; Xu et al., 2018, 2019. This character can only be coded when separate braincase ossifications can be identified.)

- - 1. At exoccipital margin
    2. Enclosed by exoccipital
    3. Unrelated to exoccipital

1. Intercalar

(Olsen, 1994; Gardiner et al., 1996; Grande, 2010; López-Arbarello, 2012; Cavin et al., 2013; Cavin et al., 2013; Xu et al., 2014b, 2018, 2019; Xu and Shen, 2015; Xu and Zhao, 2016; Deesri et al., 2014, 2016; Giles et al., 2017; López-Arbarello and Sferco, 2018. This character can only be coded when separate braincase ossifications can be identified.)

- - 1. Present
    2. Absent

1. Intercalar contributing to vagal foramen
2. Present
3. Absent
4. Membranous outgrowth of intercalar

(Gardiner et al., 1996; Hurley et al., 2007; Giles et al., 2017; López-Arbarello and Sferco, 2018; Xu et al., 2018, 2019. This character can only be coded when separate braincase ossifications can be identified.)

- - 1. Absent
    2. Present

1. Basisphenoid

(Cavin, 2010; López-Arbarello, 2012; Cavin et al., 2013; Deesri et al., 2014, 2016; Giles et al., 2017; López-Arbarello and Sferco, 2018; Xu et al., 2018, 2019)

- - 1. Present
    2. Absent or very reduced

1. Opisthotic

(Grande and Bemis, 1998; Cavin and Suteethorn, 2006; Hurley et al., 2007; Grande, 2010; López-Arbarello, 2012; Cavin et al., 2013; Deesri et al., 2014, 2016; Xu et al., 2014b, 2018, 2019; Giles et al., 2017; Ma and Xu, 2017; Xu and Ma, 2018; López-Arbarello and Sferco, 2018. This character can only be coded when separate braincase ossifications can be identified.)

- - 1. Present
    2. Absent

1. Pterotic

(Grande and Bemis, 1998; Xu and Wu, 2012; Brito and Alvarado-Ortega, 2013; Xu et al., 2014b, 2018, 2019; Xu and Shen, 2015; Sun et al., 2017; Giles et al., 2017; Ma and Xu, 2017; Xu and Ma, 2018. This character can only be coded when separate braincase ossifications can be identified.)

- - 1. Present
    2. Absent

1. Supraotic

(Gardiner et al., 1996; López-Arbarello and Sferco, 2018; Xu et al., 2018, 2019. This character can only be coded when separate braincase ossifications can be identified.)

1. Absent
2. Present
3. Ethmoid ossification

(Grande, 2010; Xu et al., 2014b, 2018, 2019; Xu and Shen, 2015; Sun et al., 2017. This character can only be coded when separate braincase ossifications can be identified.)

1. Present
2. Absent
3. Anterior extent of parasphenoid tooth patch

(Grande and Bemis, 1998; Xu et al., 2014b, 2018, 2019; Xu and Shen, 2015; Ma and Xu, 2017; Xu and Ma, 2018; López-Arbarello and Sferco, 2018)

- - 1. Extends well anterior to lateral ascending arms of parasphenoid
    2. Short, does not extend anterior to lateral ascending arms
    3. Parasphenoid tooth patch absent

1. Parasphenoid

(Coates, 1999; Gardiner et al., 2005; Friedman, 2007; Xu and Gao, 2011; Xu et al., 2012, 2014a,b, 2018, 2019; Xu and Zhao, 2016; Giles et al., 2017; López-Arbarello and Sferco, 2018)

- - 1. Terminates at/anterior to ventral otic fissure
    2. Extends across ventral otic fissure
    3. Extends to basioccipital

1. Intercalar/parasphenoid contact

(Gardiner et al., 1996; Xu et al., 2014b, 2018, 2019; Xu and Shen, 2015; Sun et al., 2017; Xu and Ma, 2018. This character can only be coded when separate braincase ossifications can be identified.)

- - 1. Absent
    2. Present

1. Internal carotid foramen on parasphenoid

(Gardiner et al., 1996; Hurley et al., 2007; Xu and Wu, 2012; Xu et al., 2012, 2014a,b, 2015, 2018, 2019; Xu and Shen, 2015; Xu and Zhao, 2016; Giles et al., 2017; Ma and Xu, 2017; Xu and Ma, 2018)

- - 1. Absent
    2. Present

1. Efferent pseudobranchial foramen on parasphenoid

(Gardiner et al., 1996; Hurley et al., 2007; Xu and Wu, 2012; Xu et al., 2012, 2014a,b, 2015, 2018, 2019; Xu and Shen, 2015; Xu and Zhao, 2016; Sun et al., 2017; Giles et al., 2017; Ma and Xu, 2017; Xu and Ma, 2018)

- - 1. Absent
    2. Present

1. Parasphenoid ascending process

(Gardiner et al., 1996; Xu et al., 2018, 2019)

1. Does not contact sphenotic
2. Contacts sphenotic
3. Sphenotic with small dermal component

(Grande, 2010; Xu and Wu, 2012; Xu et al., 2012, 2014b, 2015, 2018, 2019; Xu and Shen, 2015; Sun et al., 2017; Giles et al., 2017; Ma and Xu, 2017; Xu and Ma, 2018; López-Arbarello and Sferco, 2018)

- - 1. Absent
    2. Present

1. Posterior myodome

(Gardiner and Schaeffer, 1989; Gardiner et al., 1996; Hurley et al., 2007; Coates, 1999; Xu and Gao, 2011; Xu and Wu, 2012; Xu et al., 2012, 2014a,b, 2015, 2018, 2019; López-Arbarello, 2012; Xu and Zhao, 2016; Giles et al., 2017; López-Arbarello and Sferco, 2018)

- - 1. Present
    2. Absent

1. Lateral process of basioccipital

(Grande, 2010, Cavin et al., 2013; Deesri et al., 2014, 2016; Xu et al., 2018, 2019)

- - 1. Absent
    2. Present

1. Posterior extent of exoccipitals in adult-sized individuals

(Grande and Bemis, 1998; Xu et al., 2014b, 2018, 2019; Xu and Shen, 2015; Sun et al., 2017; Ma and Xu, 2017; Xu and Ma, 2018; Ebert, 2018)

- - 1. Reaches posterior margin of occiput
    2. Does not reach posterior margin of occiput

1. Supraoccipital bone

(Grande, 2010; López-Arbarello, 2012; Arratia, 2013; Cavin et al., 2013; Deesri et al., 2014, 2016; Xu et al., 2014b, 2018, 2019; Xu and Shen, 2015; Sun et al., 2017; Giles et al., 2017; Xu and Ma, 2018; López-Arbarello and Sferco, 2018. This character can only be coded when separate braincase ossifications can be identified.)

- - 1. Absent
    2. Present

**Snout and skull roof**

1. Elongation of the rostral region anterior to the lower jaw symphysis

(Grande, 2010; López-Arbarello, 2012; Cavin et al., 2013; Deesri et al., 2014, 2016; López-Arbarello and Sferco, 2018; Xu et al., 2018, 2019)

1. Extends anterior to the dentary symphysis by less than 20% of mandibular length
2. Extends well anterior to the dentary symphysis by more than 50% of mandibular length
3. Rostral bone

(Gardiner et al., 1996; Grande and Bemis, 1998; Xu and Wu, 2012; Xu et al., 2012, 2014a,b, 2018, 2019; Brito and Alvarado-Ortega, 2013; Xu and Shen, 2015; Sun et al., 2017; Giles et al., 2017; Ma and Xu, 2017; Xu and Ma, 2018)

- - - 1. A deep cap on tip of snout
      2. Of moderate size, sub-circular
      3. Much reduced, sub-triangular, elongate, V-shaped or tube-like
      4. Lost as an autogenous bone

1. Dermal bones of the skull ornamented with firmly anchored, pointed conical denticles

(Grande, 2010; López-Arbarello, 2012; Cavin et al., 2013; Deesri et al., 2016; López-Arbarello and Sferco, 2018; Xu et al., 2018, 2019)

- - - 1. Absent
      2. Present

1. Parietal length

(Grande and Bemis, 1998; Xu et al., 2014b, 2018, 2019; Xu and Shen, 2015; Sun et al., 2017; Ma and Xu, 2017; Xu and Ma, 2018; Ebert, 2018; López-Arbarello and Sferco, 2018)

1. Short, with a width-to-length ratio range well exceeding 0.90
2. Relatively long, with a width-to-length ratio range not exceeding 0.90
3. Number of parietal bones

(Grande and Bemis, 1998; Xu et al., 2014; Xu and Shen, 2015; Sun et al., 2017; Ma and Xu, 2017; Xu and Ma, 2018)

1. Paired parietals normally present
2. Only a single median parietal
3. Paired frontals fused into a median bone:

(López-Arbarello et al., 2019)

- 1. Absent
  2. Present

1. Ratio of frontal/parietal length

(Modified from López-Arbarello, 2012; Cavin et al., 2013; Deesri et al., 2016; López-Arbarello and Wencker, 2016; Sun et al., 2017; López-Arbarello and Sferco, 2018; Xu et al., 2018, 2019)

1. 1.5 or more
2. Less than 1.5
3. Frontal width in adult-sized individuals

(Modified from Grande and Bemis, 1998; López-Arbarello, 2012; Xu et al., 2014b, 2018, 2019; Xu and Shen, 2015; López-Arbarello and Wencker, 2016; Sun et al., 2017; Ma and Xu, 2017; Xu and Ma, 2018; López-Arbarello and Sferco, 2018)

1. Relatively wide, with a width-to-length ratio of 0.22 to 0.65
2. Relatively narrow, with a width-to-length ratio of no more than 0.21
3. Pre-orbital length equal to or longer than orbital length

(Xu et al., 2019)

1. Absent
2. Present
3. Shape of dermopterotic (=supratemporal+intertemporal)

(Grande and Bemis, 1998; Xu et al., 2014b, 2018, 2019; Xu and Shen, 2015; Sun et al., 2017; Ma and Xu, 2017; Xu and Ma, 2018)

1. Greatly widened posteriorly and tapered anteriorly
2. Subrectangular, not substantially tapered anteriorly or widened posteriorly
3. Dermopterotic length to parietal length

(Modified from Gardiner et al., 1996; Grande and Bemis, 1998; Xu et al., 2014b, 2018, 2019; Xu and Shen, 2015; López-Arbarello and Wencker, 2016; Sun et al., 2017; Ma and Xu, 2017; Xu and Ma, 2018)

1. Dermopterotic significantly longer
2. Lengths about equivalent
3. Dermopterotic shorter than parietal
4. Number of extrascapular bones

(Grande and Bemis, 1998; Xu et al., 2014b, 2018; Xu and Shen, 2015; Sun et al., 2017; Ma and Xu, 2017; Xu and Ma, 2018; López-Arbarello and Sferco, 2018)

1. One pair
2. Two pairs
3. More than two pairs
4. Extrascapulars separated by posterior extensions of parietals

(López-Arbarello, 2012; López-Arbarello and Wencker, 2016; Sun et al., 2017; López-Arbarello and Sferco, 2018; Xu et al., 2018, 2019)

1. Absent
2. Present
3. Long and narrow anterorbital portion of frontal

(Modified from López-Arbarello, 2012; López-Arbarello and Wencker, 2016; Sun et al., 2017; López-Arbarello and Sferco, 2018; Xu et al., 2018, 2019)

1. Absent
2. Present
3. Dermopterotic ribs

(Grande and Bemis, 1998; Xu et al., 2014; Xu and Shen, 2015; Sun et al., 2017; Ma and Xu, 2017; Xu and Ma, 2018)

1. Absent
2. Present
3. Rostral/frontal contact

(Grande and Bemis, 1998; Xu et al., 2014a,b, 2018, 2019; Xu and Shen, 2015; Sun et al., 2017; Ma and Xu, 2017; Xu and Ma, 2018)

1. Present
2. Absent
3. Contact relationships of nasals

(Arratia, 2013; Ma and Xu, 2017; Giles et al., 2017; Xu and Ma, 2018; López-Arbarello and Sferco, 2018; Xu et al., 2018, 2019)

1. Separated by rostral
2. Contacting medially or close to contact medially (not separated by other bones)
3. Separated by frontals
4. Separated by mesethmoid
5. Nasal contributing to orbital margin

(Xu and Wu, 2012; Xu et al., 2014a, 2018, 2019; Xu and Zhao, 2016; Giles et al., 2017; López-Arbarello and Sferco, 2018)

1. Present
2. Absent
3. Frontal contributing to orbital margin

(Xu et al., 2018, 2019)

1. Absent
2. Present
3. Commissure between right and left supraorbital canal within frontal

(Grande, 2010; Cavin et al., 2013; Deesri et al., 2016; Xu et al., 2018, 2019)

1. Absent
2. Present
3. Parietal portion of the supraorbital sensory canal

(Cavin, 2010; López-Arbarello, 2012; Cavin et al., 2013; Deesri et al., 2016; Ma and Xu, 2017; Sun et al., 2017; Xu and Ma, 2018; Xu et al., 2018, 2019)

- - 1. Present
    2. Absent

1. Junction of supraorbital canal with infraorbital canal exclusively in the dermopterotic

(Grande, 2010; Ma and Xu, 2017; Xu and Ma, 2018; Xu et al., 2018, 2019)

1. Absent
2. Present
3. Interfrontal fontanelle in adult-sized individuals

(Grande and Bemis, 1998; Xu et al., 2014; Xu and Shen, 2015; Sun et al., 2017; Ma and Xu, 2017; Xu and Ma, 2018; López-Arbarello and Sferco, 2018)

1. Absent, frontals sutured to each other medially for their entire length
2. Frontals separated for about one-half their length or more by a fontanelle

**Circumorbital bones**

1. Tube-like canal bearing anterior arm on the antorbital bone.

(Grande, 2010; Xu and Wu, 2012; Brito and Alvarado-Ortega, 2013; Xu et al., 2014b, 2018, 2019; Xu and Shen, 2015; Ma and Xu, 2017; Sun et al., 2017; Giles et al., 2017; Xu and Ma, 2018; López-Arbarello and Sferco, 2018)

1. Absent
2. Present
3. Supraorbital

(Modified from Grande and Bemis, 1998; Brito and Alvarado-Ortega, 2013; Xu et al., 2014a,b, 2018, 2019; Xu and Shen, 2015; Sun et al., 2017; Giles et al., 2017; Ma and Xu, 2017; Xu and Ma, 2018; López-Arbarello and Sferco, 2018)

- 1. Absent
  2. Present

1. Number of supraorbital bones

(Modified from Grande and Bemis, 1998; Brito and Alvarado-Ortega, 2013; Xu et al., 2014a,b, 2018, 2019; Xu and Shen, 2015; Sun et al., 2017; Giles et al., 2017; Ma and Xu, 2017; Xu and Ma, 2018; Ebert, 2018; López-Arbarello and Sferco, 2018.)

1. Three or more
2. Two
3. Single
4. Depth of supraorbital bones relative to orbit

(Modified from López-Arbarello, 2012; Xu et al., 2019)

- - - 1. Dorsoventrally short
      2. Deeper than orbital radius

1. Supraorbital/antorbital contact

(Xu et al., 2018, 2019)

- - - 1. Absent
      2. Present

1. Antorbital/frontal contact

(Xu et al., 2018, 2019)

- - - 1. Absent
      2. Present

1. Infraorbital/dermopterotic contact

(Xu et al., 2018, 2019)

- - - 1. Absent
      2. Present

1. Anterior infraorbitals

(Cavin and Suteethorn, 2006; Grande, 2010; Xu and Wu, 2012; López-Arbarello, 2012; Xu et al. 2014b, 2015, 2018, 2019; Xu and Zhao, 2016; Giles et al., 2017; Ma and Xu, 2017; Xu and Ma, 2018; López-Arbarello and Sferco, 2018. The ‘anterior infraorbitals’ refer to the infraorbital bones that locate posterior to the antorbital and do not contribute to the orbital margin (Wenz, 1999; López-Arbarello, 2012))

1. Absent
2. Present
3. Number of anterior infraorbitals

(Cavin and Suteethorn, 2006; Grande, 2010; Xu and Wu, 2012; López-Arbarello, 2012; Xu et al. 2014b, 2015, 2018, 2019; Xu and Zhao, 2016; Giles et al., 2017; Ma and Xu, 2017; Xu and Ma, 2018; López-Arbarello and Sferco, 2018)

- - 1. One or two
    2. Three
    3. Four or more

1. Ratio of depth/length of the first anterior infraorbital

(Xu et al., 2019)

1. No more than 1
2. 1.1–2.0
3. ≥2.5
4. Contact relationships of the anterior-most supraorbital with anterior infraorbitals

(Xu et al., 2018, 2019)

1. Supraorbital does not contact anterior infraorbitals
2. Supraorbital contacts a single anterior infraorbital
3. Supraorbital contacts two or more anterior infraorbitals
4. Contact relationships of the frontal with anterior infraorbitals

(Xu et al., 2018, 2019)

1. frontal does not contact anterior infraorbitals
2. frontal contacts anterior infraorbitals
3. Contact relationships of infraorbital bone at posteroventral corner of orbit with preopercle

(Modified from López-Arbarello, 2012; Xu and Ma, 2018; López-Arbarello and Sferco, 2018; Xu et al., 2018, 2019)

- - 1. Absent
    2. Present

1. Posterior notch of second infraorbital for supramaxilla

(Xu et al., 2014b, 2018, 2019; Xu and Shen, 2015; Sun et al., 2017)

1. Absent
2. Present
3. Antorbital contributing to orbital margin

(Xu et al., 2012, 2014b, 2018, 2019; Xu and Shen, 2015; Xu and Zhao, 2016; Sun et al., 2017; López-Arbarello and Sferco, 2018)

1. Present
2. Absent
3. Lower margin of last infraorbital inclined posterodorsally

(Gardiner et al., 1996; Alvarado-Ortega and Espinosa-Arrubarrena, 2008; Xu et al., 2014b, 2018, 2019; Xu and Shen, 2015; Ma and Xu, 2017; Xu and Ma, 2018)

1. Absent
2. Present
3. Size of last infraorbital

(Modified from Grande and Bemis, 1998; Xu et al., 2018, 2019)

1. Small, relatively narrow
2. Large, posteriorly expanded
3. Number of infraorbitals between antorbital and dermosphenotic

(Gardiner and Schaeffer, 1989; Xu et al., 2012, 2014b, 2018; Xu and Shen, 2015; Ma and Xu, 2017; Xu and Ma, 2018)

1. Two or three
2. Four or five
3. Six or more
4. Number of the infraorbital bones between the Lacrimal and the infraorbital at the posteroventral corner of the orbit

(Modified from Gardiner et al., 1996; Xu et al., 2018, 2019)

1. Zero
2. One
3. Two to four
4. Number of postinfraorbital bones
   1. Two
   2. Three or more
5. Depth of infraorbital bones at middle portion of the orbit

(Xu et al., 2019)

- - - 1. Dorsoventrally short
      2. Deeper than the orbital radius

1. Lacrimal

(Modified from Grande and Bemis, 1998; Xu et al., 2014b, 2018, 2019; Xu and Shen, 2015; Sun et al., 2017; Ma and Xu, 2017; Xu and Ma, 2018)

- - 1. Longer than deep
    2. Deeper than long

1. Lacrimal relative to orbit in size

(Modified from Grande and Bemis, 1998; Xu et al., 2014b, 2018, 2019; Xu and Shen, 2015; Sun et al., 2017; Ma and Xu, 2017; Xu and Ma, 2018)

1. Significantly smaller than orbit
2. Nearly equal to orbit in size
3. Contact relationships of lacrimal with supraorbital

(Xu et al., 2019)

1. Well separated
2. Closely related or firmly contact
3. Inner orbital flange of dermosphenotic

(Grande and Bemis, 1998; Brito and Alvarado-Ortega, 2013; Xu et al., 2014b, 2018, 2019; Xu and Shen, 2015; Sun et al., 2017; Ma and Xu, 2017; Xu and Ma, 2018)

1. Smooth, without sensory canal
2. Bearing sensory canal tube
3. Dermosphenotic participation in orbital margin

(Grande and Bemis, 1998; Xu et al., 2014b, 2018, 2019; Xu and Shen, 2015; Sun et al., 2017; Ma and Xu, 2017; Xu and Ma, 2018; López-Arbarello and Sferco, 2018)

- - 1. Dermosphenotic reaches orbital margin
    2. Dermosphenotic does not reach orbital margin

1. Sphenotic with a relatively large exposed dermal component nearly reaching the orbital margin

(Xu et al., 2018, 2019)

1. Absent
2. Present
3. Dermosphenotic bone attachment to skull roof in adult-sized individuals

(Grande and Bemis, 1998; Brito and Alvarado-Ortega, 2013; Xu et al., 2014b, 2018, 2019; Xu and Shen, 2015; Sun et al., 2017; Ma and Xu, 2017; Xu and Ma, 2018; López-Arbarello and Sferco, 2018. The states of *Subortichthys*, *Asialepidotus*, *Panxianichthys* and *Robustichthys* were changed from “1” to “0” in the data matrix.)

1. Loosely attached on the skull roof or hinged to the side of skull roof
2. Firmly sutured into skull roof, forming part of it
3. Dermosphenotic/sphenotic association

(Grande, 2010; López-Arbarello and Sferco, 2018; Xu et al., 2018, 2019)

- - 1. Closely associated with each other (i.e. contacting or fused to each other)
    2. Not in contact with each other

1. Position of dermosphenotic
2. Dermosphenotic extending well below dermopterotic
3. Dermosphenotic located at same horizontal level of dermopterotic
4. Sclerotic ring ossification

(Grande and Bemis, 1998; Xu et al., 2014b, 2018, 2019; Xu and Shen, 2015; Sun et al., 2017; Ma and Xu, 2017; Xu and Ma, 2018; Ebert, 2018; López-Arbarello and Sferco, 2018)

1. Present
2. Absent
3. Suborbital bones

(Grande and Bemis, 1998; Xu and Wu, 2012; Xu et al., 2014a,b, 2018, 2019; Xu and Shen, 2015; Sun et al., 2017; Giles et al., 2017; Ma and Xu, 2017; Xu and Ma, 2018; Ebert, 2018; López-Arbarello and Sferco, 2018)

- - 1. Present
    2. Absent

1. Number of suborbital bones

(Modified from Xu and Gao, 2011; Xu et al., 2014b, 2018, 2019; López-Arbarello and Wencker, 2016; Giles et al., 2017; López-Arbarello and Sferco, 2018)

- - 1. One
    2. Two to six
    3. Seven or more

1. Dermohyal

(Grande and Bemis, 1998; Xu and Gao, 2011; Xu et al., 2014a,b, 2015, 2019)

- - 1. Present
    2. Absent

1. Suborbital bones extending anteriorly below the orbit

(López-Arbarello, 2012; López-Arbarello and Sferco, 2018; Xu et al., 2018, 2019)

- - - 1. Absent
      2. Present

1. Suborbitals separated by posteroventral infraorbital into dorsal and ventral portions

(Xu et al., 2019)

- - - 1. Absent
      2. Present

**Palatoquadrate, hyoid and branchial arches**

1. Vomer in adults

(Brito and Alvarado-Ortega, 2013; Xu et al., 2014b, 2018, 2019; Xu and Shen, 2015; Xu and Ma, 2016; Sun et al., 2017; Giles et al., 2017; Ma and Xu, 2017; Xu and Ma, 2018; López-Arbarello and Sferco, 2018)

- - 1. Paired
    2. Median

1. Autopalatine

(Grande, 2010; López-Arbarello, 2012; López-Arbarello and Wencker, 2016; Sun et al., 2017; López-Arbarello and Sferco, 2018; Xu et al., 2018, 2019)

- - 1. Present
    2. Absent

1. Endopterygoid/dermopalatine association

(Grande, 2010; López-Arbarello and Sferco, 2018; Xu et al., 2018, 2019)

1. Endopterygoid sutured to dermopalatine anteriorly
2. Endopterygoid not in contact with any dermopalatine
3. Length of ectopterygoid relative to endopterygoid

(Grande, 2010; Cavin et al., 2013; Deesri et al., 2016; Xu et al., 2018, 2019)

- 1. Twice the length of the endopterygoid or less
  2. More than twice the length of the endopterygoid

1. Quadrate/metapterygoid contact or close association

(Grande, 2010; Deesri et al., 2016; López-Arbarello and Sferco, 2018; Xu et al., 2018, 2019)

1. Present
2. Absent
3. Laterally sliding articulation between metapterygoid and the parasphenoid-prootic process

(Grande, 2010; Cavin et al., 2013; Deesri et al., 2016; López-Arbarello and Sferco, 2018; Xu et al., 2018, 2019)

1. Absent
2. Present
3. Ectopterygoid participation in palatal surface area

(Grande, 2010; López-Arbarello, 2012; López-Arbarello and Sferco, 2018; Xu et al., 2018, 2019)

- - 1. Ectopterygoid form half or less of the palatal region
    2. Ectopterygoid forms the majority of the palatal region

1. Part of dorsal surface of ectopterygoid ornamented and forming part of skull roof

(Grande, 2010; López-Arbarello, 2012; Cavin et al., 2013; Deesri et al., 2016; López-Arbarello and Sferco, 2018; Xu et al., 2018, 2019)

- - 1. Absent
    2. Present

1. Urohyal

(Grande, 2010; López-Arbarello and Sferco, 2018; Xu et al., 2018, 2019)

1. Absent
2. Present
3. Basihyal tooth plate (‘tongue bone’) consisting of a mosaic of bony plates (‘entoglossals’). (Grande, 2010; Cavin et al., 2013; Deesri et al., 2016; Xu et al., 2018, 2019)
4. Absent
5. Present
6. Elongated posteroventral process of quadrate

(Arratia, 2013; Xu et al., 2014b, 2018; Xu and Shen, 2015; Xu and Zhao, 2016; Xu et al., 2018, 2019)

1. Absent
2. Present
3. Quadrate

(Modified from Cavin, 2010; Cavin et al., 2013; Deesri et al., 2014, 2016; López-Arbarello and Sferco, 2018; Xu et al., 2018, 2019)

1. Not exposed (except condyle)
2. Partly or fully exposed
3. Quadrate almost fully covered by infraorbital(s)

(Xu et al., 2018, 2019)

- - 1. Absent
    2. Present

1. Quadrate partly or almost fully covered by maxilla
   - 1. Absent
     2. Present
2. Symplectic

(Gardiner and Schaeffer, 1989; Coates 1999; Hurley et al., 2007; Xu and Zhao, 2016; Giles et al., 2017; López-Arbarello and Sferco, 2018; Xu et al., 2018, 2019. The ossification that contacts the hyomandibula (and typically the quadrate), but does not articulate with the ceratohyal, is termed the symplectic. The ossification that forms an intermediary between the hyomandibula and ceratohyal is termed the interhyal. We follow Gardiner et al. (1996) in identifying the ‘interhyal’ of *Pteronisculus*, *Boreosomus*, and *Pycholepis* (Nielsen, 1942, 1949; Véran, 1981, 1988) as a posterior ceratohyal, and the ‘symplectic’ of these taxa as an interhyal.)

1. Absent
2. Present
3. Symplectic shape

(Grande, 2010; Cavin et al., 2013; Deesri et al., 2016; Giles et al., 2017; López-Arbarello and Sferco, 2018; Xu et al., 2018, 2019)

1. Slightly curved tube or splint
2. Hourglass-shaped or hatchet-shaped
3. L-shaped
4. Position of the quadrate-mandibular articulation

(Modified from Grande, 2010; Xu et al., 2014b, 2018, 2019; Xu and Shen, 2015; Sun et al., 2017; Ma and Xu, 2017; Xu and Ma, 2018; López-Arbarello and Sferco, 2018)

- - 1. Under the posterior border of the orbit or posterior to the orbit
    2. Under the orbit
    3. Under the anterior border of the orbit or anterior to the orbit

1. Quadratojugal

(Modified from Grande, 2010; Cavin, 2010; Brito and Alvarado-Ortega, 2013; López-Arbarello, 2012; Cavin et al., 2013; Deesri et al., 2014, 2016; Xu and Zhao, 2016; Xu and Ma, 2016; Sun et al., 2017; Ma and Xu, 2017; Xu and Ma, 2018, 2019; López-Arbarello and Sferco, 2018; Xu et al., 2018)

- - 1. Present
    2. Absent or fused to quadrate

1. Shape of quadratojugal

(Gardiner et al., 1996; Hurley et al., 2007; López-Arbarello and Wencker, 2016; Xu et al., 2018, 2019)

- - 1. Plate-like
    2. Splint-like
    3. Reduced to a small flange of bone on quadrate

1. Symplectic/quadrate contact

(Grande, 2010; Cavin et al., 2013; Xu et al., 2014b, 2018, 2019; Xu and Shen, 2015; Deesri et al., 2016; Sun et al., 2017; Ma and Xu, 2017; Xu and Ma, 2018; Ebert, 2018; López-Arbarello and Sferco, 2018)

- - 1. Present
    2. Absent

1. Symplectic involvement in jaw joint

(Grande and Bemis, 1998; Grande 2010; Xu and Wu, 2012; Brito and Alvarado-Ortega, 2013; Xu et al., 2014b, 2015, 2018, 2019; Xu and Shen, 2015; Sun et al., 2017; Ma and Xu, 2017; Giles et al., 2017; Xu and Ma, 2018)

- - - 1. Absent
      2. Present

1. Elongation of opercular process of hyomandibula

(Grande and Bemis, 1998; Xu et al., 2014; Xu and Shen, 2015; Sun et al., 2017; Ma and Xu, 2017; Xu and Ma, 2018)

1. Absent
2. Present
3. Preopercular process at the posterior margin of hyomandibula

(Arratia, 2013; López-Arbarello and Sferco, 2018; Xu et al., 2018, 2019)

1. Absent
2. Present
3. Number of hypobranchials

(Grande, 2010; Xu and Wu, 2012; Brito and Alvarado-Ortega, 2013; Xu et al., 2014a,b, 2018, 2019; Xu and Shen, 2015; Xu and Ma, 2016; Xu and Zhao, 2016; Sun et al., 2017; Giles et al., 2017; López-Arbarello and Sferco, 2018)

1. Four
2. Three

**Jaws**

1. Olfactory nerve pierces premaxilla

(Grande, 2010; Xu and Wu, 2012; Cavin et al., 2013; Xu et al., 2014a,b, 2015, 2018, 2019; Xu and Shen, 2015; Xu and Zhao, 2016; Deesri et al., 2016; Sun et al., 2017; Giles et al., 2017; Xu and Ma, 2018)

- - 1. Absent
    2. Present

1. Nasal process of premaxilla

(Gardiner and Schaeffer, 1989; Gardiner et al., 1996; Gardiner et al., 2005; Cavin and Suteethorn, 2006; Hurley et al., 2007; Grande, 2010; López-Arbarello, 2011; Xu and Wu, 2012; Cavin et al., 2013; Xu et al., 2014b, 2018, 2019; Xu and Shen, 2015; Xu and Zhao, 2016; Deesri et al., 2016; Giles et al., 2017; Xu and Ma, 2018)

- - 1. Absent
    2. Present

1. Length of the nasal process of premaxilla

(Grande, 2010; Xu and Wu, 2012; Xu et al., 2014b, 2018, 2019; Xu and Shen, 2015; Sun et al., 2017; Xu and Ma, 2018; López-Arbarello and Sferco, 2018)

1. Relatively short, not sutured to frontals
2. Deep, tightly sutured to frontals
3. Nasal process of premaxilla forms much of the ornamented dermal roof in the snout region

(Grande, 2010; López-Arbarello, 2012; Xu and Ma, 2018; Xu et al., 2018, 2019)

- - 1. Absent
    2. Present

1. Supraorbital canal incorporated into premaxilla

(Grande, 2010; López-Arbarello, 2012; Cavin et al., 2013; Deesri et al., 2014, 2016; Xu and Ma, 2018; Xu et al., 2018, 2019)

- - 1. Absent
    2. Present

1. Premaxillary tooth row curves anteriorly at symphysis and laterally onto projecting horns as it nears frontal

(Grande, 2010; Cavin et al., 2013; Deesri et al., 2014, 2016; Xu et al., 2018, 2019)

1. Absent
2. Present
3. Mobile maxilla in cheek

(Coates, 1999; Grande, 2010; Brito and Alvarado-Ortega, 2013; Xu et al., 2012, 2014b, 2018, 2019; Xu and Shen, 2015; Sun et al., 2017; Giles et al., 2017)

1. Absent
2. Present
3. Maxilla extremely slender and rod-like

(Grande and Bemis, 1998; Xu et al., 2014b, 2018, 2019; Xu and Shen, 2015; Sun et al., 2017; Ma and Xu, 2017; Xu and Ma, 2018, 2019)

1. Absent
2. Present
3. Peg-like anterior process of maxilla

(Grande, 2010; Xu and Wu, 2012; Cavin et al., 2013; Xu et al., 2014b, 2018, 2019; Deesri et al., 2016; Giles et al., 2017)

1. Absent
2. Present
3. Lateral line canal in maxilla

(Gardiner et al., 1996; Grande and Bemis, 1998; Brito and Alvarado-Ortega, 2013; Xu et al., 2014b, 2018; Xu and Shen, 2015; Sun et al., 2017; Xu and Ma, 2018; López-Arbarello and Sferco, 2018)

1. Absent
2. Present
3. Posterior margin of maxilla

(Grande and Bemis, 1998; Xu and Wu, 2012; Xu et al., 2014b, 2015, 2018, 2019; Brito and Alvarado-Ortega, 2013; Arratia, 2013; Xu and Zhao, 2016; Giles et al., 2017; Ma and Xu, 2017; Xu and Ma, 2018)

1. Convexly rounded or straight
2. Excavated
3. Size of postmaxillary process under postmaxillary notch

(Grande and Bemis, 1998; Xu et al., 2014; Xu and Shen, 2015; Ma and Xu, 2017; Xu and Ma, 2018; López-Arbarello and Sferco, 2018)

1. Relatively small and short
2. Thick and elongate
3. Coronoid process

(Gardiner and Schaeffer, 1989; Xu et al., 2014b, 2018, 2019; Xu and Shen, 2015; Sun et al., 2017; Giles et al., 2017; Xu and Ma, 2018; López-Arbarello and Sferco, 2018)

1. Absent
2. Present
3. Composition of coronoid process

(Xu et al., 2018, 2019)

1. Formed by dentary and supra-angular
2. By dentary and angular
3. Mainly by dentary
4. Lacrimomaxillary bones

(Grande, 2010; Xu et al., 2018, 2019)

- - 1. Absent
    2. Present

1. Posterior end of maxilla relative to coronoid process

(López-Arbarello, 2012; López-Arbarello and Sferco, 2018; Xu et al., 2018, 2019)

- - 1. At the level of the coronoid process
    2. Anterior to coronoid process
    3. Well posterior to the coronoid process

1. Posterior end of maxilla relative to orbit

(Modified from Gardiner et al., 1996; López-Arbarello and Sferco, 2018; Xu et al., 2018, 2019)

- - 1. Posterior to the orbit
    2. Below the orbit
    3. Anterior to the orbit

1. Oral margin of maxilla

(Xu et al., 2018, 2019)

- - 1. Slightly concave or straight
    2. Convex

1. Depth of maxilla

(Modified from López-Arbarello, 2012; López-Arbarello and Sferco, 2018; Xu et al., 2018, 2019)

- - 1. Shallow, < 0.5 of its length
    2. Deep, > 0.5 of its length

1. Suborbital/maxilla contact

(Xu et al., 2012, 2014b, 2015, 2018, 2019; Xu and Ma, 2016)

- - 1. Present
    2. Absent

1. Supramaxilla

(Gardiner and Schaeffer, 1989; Grande and Bemis, 1998; Xu and Wu, 2012; Xu et al., 2014b, 2018, 2019; Xu and Shen, 2015; Xu and Ma, 2016; Sun et al., 2017)

1. Absent
2. Present
3. Number of supramaxilla

(Grande and Bemis, 1998; Brito and Alvarado-Ortega, 2013; Xu et al., 2014b, 2018, 2019; Xu and Shen, 2015; Sun et al., 2017; Ma and Xu, 2017; Xu and Ma, 2018)

1. one
2. two
3. Dorsal process of maxilla for single supramaxilla

(Modified from Xu et al., 2018, 2019)

- - 1. Well developed
    2. Much reduced or absent

1. Shape of supramaxilla

(Grande and Bemis, 1998; Xu et al., 2014; Xu and Shen, 2015; Sun et al., 2017; Xu and Ma, 2018)

1. Elongate
2. Extremely deep, shaped like a rounded triangle
3. Maxilla/preopercle contact:
   1. Present
   2. Absent
4. Teeth on maxilla

(López-Arbarello, 2012; Xu et al., 2014b, 2015, 2018, 2019; Xu and Ma, 2016; Xu and Zhao, 2016; Giles et al., 2017)

- - 1. Present
    2. Absent

1. Plicidentine tooth structure

(Grande, 2010; López-Arbarello and Sferco, 2018; Xu et al., 2018, 2019)

- - 1. Absent
    2. Present

1. Coronoid bones

(Grande, 2010; Cavin et al., 2013; Deesri et al., 2016; López-Arbarello and Sferco, 2018; Xu et al., 2018, 2019)

1. Present as separate ossifications
2. Absent
3. Teeth on anterior coronoids

(Grande and Bemis, 1998; Xu et al., 2014b, 2018, 2019; Xu and Shen, 2015; Sun et al., 2017; Ma and Xu, 2017; Xu and Ma, 2018; López-Arbarello and Sferco, 2018)

1. Conical or pointed pencil like
2. Styliform, with broadly rounded or flattened tips
3. Number of tooth rows on coronoids

(Grande and Bemis, 1998; Xu et al., 2014b, 2018, 2019; Xu and Shen, 2015; Sun et al., 2017; Ma and Xu, 2017; Xu and Ma, 2018)

1. Two or more rows for at least part of one or more coronoids
2. One row
3. Arrangement of vomerine teeth

(Grande and Bemis, 1998; Xu et al., 2014b, 2018; Xu and Shen, 2015; Sun et al., 2017; Ma and Xu, 2017; Xu and Ma, 2018)

1. Tooth patch with two to several rows of teeth
2. Tooth patch with only a single anterior marginal row, plus one or more teeth in a longitudinal series perpendicular to the anterior marginal row
3. Supra-angular

(Grande, 2010; Brito and Alvarado-Ortega, 2013; Cavin et al., 2013; Deesri et al., 2016; Xu et al., 2015, 2018, 2019; Xu and Ma, 2016, 2018; Ma and Xu, 2017; Xu and Ma, 2018; López-Arbarello and Sferco, 2018)

1. Present
2. Absent
3. ‘Leptolepid’ notch in ascending margin of dentary

(Arratia, 2013; Ma and Xu, 2017; Giles et al., 2017; Xu and Ma, 2018; Xu et al., 2018, 2019)

1. Absent
2. Present
3. Mobile premaxilla

(Arratia, 2013; Xu and Ma, 2016, 2018; Ma and Xu, 2017; Xu and Ma, 2018; Xu et al., 2018, 2019)

1. Absent
2. Present
3. Anterior end of first coronoid curves medially and expands broadly to a flat symphysis

(Grande, 2010; Xu et al., 2018, 2019)

1. Absent
2. Present
3. Mentomeckelian bone

(Grande, 2010; Cavin et al., 2013; Deesri et al., 2016; Xu et al., 2018, 2019)

1. Present
2. Absent
3. Type of dentary symphysis

(Grande, 2010; Xu et al., 2018, 2019)

1. Symphysis occurs between the recurved anterior ends of right and left dentary
2. Symphysis occurs along medial surface of anterior right and left dentary with anterior ends pointing anterior
3. Prearticular

(Grande, 2010; Cavin et al., 2013; Deesri et al., 2016; López-Arbarello and Sferco, 2018; Xu et al., 2018, 2019)

1. Present
2. Absent
3. Mandibular length as a percentage of head length

(Grande, 2010; Cavin et al., 2013; Deesri et al., 2016; Xu et al., 2018, 2019)

1. 44% or more
2. Less than 43%
3. Supra-angular/angular contact

(Xu et al., 2018, 2019)

- - 1. Present
    2. Absent

1. Articular ossification of lower jaw

(Grande and Bemis, 1998; Xu et al., 2014b, 2018, 2019; Xu and Shen, 2015; Sun et al., 2017; Ma and Xu, 2017; Xu and Ma, 2018; López-Arbarello and Sferco, 2018)

1. A single element, or two elements tightly sutured to each other
2. Two separate elements not in contact with each other
3. Teeth on dentary

(Xu et al., 2014b, 2018, 2019; Xu and Ma, 2016; Giles et al., 2017; López-Arbarello and Sferco, 2018)

- - 1. Present
    2. Absent

1. Tooth organization on dentary

(Grande, 2010; Cavin et al., 2013; Deesri et al., 2016; López-Arbarello and Sferco, 2018; Xu et al., 2018, 2019)

1. Dentary teeth in a single row and all of similar size
2. In addition to a single row of similar sized teeth, there is a median row of much larger fangs
3. A pavement of small similar sized teeth not in rows
4. Well-developed posteroventral process of the dentary

(Cavin, 2010; López-Arbarello and Wencker, 2016; López-Arbarello and Sferco, 2018; Xu et al., 2018, 2019)

- - 1. Absent
    2. Present

1. Morphology of caps of the jaw teeth in adult-sized individuals

(Grande and Bemis, 1998; Xu et al., 2014b, 2018, 2019; Xu and Shen, 2015; Sun et al., 2017; Ma and Xu, 2017; Xu and Ma, 2018)

1. Round in cross-section, not sharply carinate
2. Labiolingually compressed, sharply carinate (keeled)
3. Extent of teeth on dentary (excluding coronoid toothplates)

(Grande, 2010; López-Arbarello and Sferco, 2018; Xu et al., 2018, 2019)

- - 1. Tooth row extends over a third the length of dentary
    2. Tooth row is present on only the anterior one third or less of dentary

**Opercular series, branchiostegals and gular**

1. Shape of preopercle

(Grande and Bemis, 1998; Brito and Alvarado-Ortega, 2013; Xu et al., 2014b, 2018; Xu and Shen, 2015; Sun et al., 2017; Ma and Xu, 2017; Xu and Ma, 2018; López-Arbarello and Sferco, 2018)

- - 1. Dorsally expanded, without anteroventral arm
    2. Crescent-shaped
    3. L-shaped
    4. Oviod

1. Peculiar ornamentation pattern of strongly defined, converging lines on opercles in adult-sized individuals

(Grande and Bemis, 1998; Xu et al., 2014; Xu and Shen, 2015; Sun et al., 2017; Ma and Xu, 2017; Xu and Ma, 2018)

1. Absent
2. Present
3. Width of opercle

(Grande and Bemis, 1998; Xu et al., 2014b, 2018, 2019; Xu and Shen, 2015; Sun et al., 2017; Ma and Xu, 2017; Xu and Ma, 2018)

1. Narrow, with width-to-height ratio of 0.56 to 1.06
2. Wide, with width-to-height ratio in range of 1.07 to 1.39
3. Exposure of dorsal limb of preopercle

(Grande, 2010; López-Arbarello and Sferco, 2018; Xu et al., 2018, 2019)

- - 1. Mostly exposed forming a significant part of the ornamented lateral surface of the skull anterior to the opercle
    2. Entirely covered or nearly entirely covered by other dermal bones in adults

1. Preopercle/dermopterotic contact

(Xu and Ma, 2016; López-Arbarello and Sferco, 2018; Xu et al., 2018, 2019)

1. Present
2. Absent
3. Posterior border of preopercle notched ventrally

(López-Arbarello, 2012; Giles et al., 2017; Xu et al., 2018, 2019)

- - 1. Absent
    2. Present

1. Subopercle with well-developed anterodorsal process

(López-Arbarello, 2012; Giles et al., 2017; Xu et al., 2018, 2019)

- - 1. Absent
    2. Present

1. Depth of ascending process of the subopercle

(López-Arbarello, 2012; López-Arbarello and Sferco, 2018; Xu et al., 2018, 2019)

- - 1. Less than or equal to half of the length of the opercle
    2. More than half of the length of the dorsal border of the bone

1. Interopercle

(Gardiner and Schaeffer, 1989; Xu and Gao, 2011; Xu et al., 2014b, 2015, 2018; Gardiner and Schaeffer, 1989; Olsen and McCune, 1991; Gardiner et al., 1996; Gardiner et al., 2005; Cavin and Suteethorn, 2006; Hurley et al., 2007; López-Arbarello, 2012; Xu and Zhao, 2016; Giles et al., 2017)

- - 1. Absent
    2. Present

1. Shape of interopercle
   - - - 1. Triangular
         2. Nearly trapezoidal
2. Position of anterior tip of the interopercle

(Olsen and McCune, 1991; López-Arbarello and Sferco, 2018; Xu et al., 2018)

- - 1. Close to the lower jaw
    2. Far away from the lower jaw

1. Lateral gulars

(Xu et al., 2014a,b, 2018; Giles et al., 2017. The anterior most ‘fifth branchiostegal ray’ of the ‘*Perleidus*’ species from Madagascar is reinterpreted as the lateral gular because it has pit-lines.)

1. Present
2. Absent
3. Median gular

(Grande and Bemis, 1998; Brito and Alvarado-Ortega, 2013; Xu et al., 2014a,b, 2018; Xu and Shen, 2015; Sun et al., 2017; Ma and Xu, 2017; Xu and Ma, 2018)

- - 1. Present
    2. Absent

1. Ventral transverse ridge of median gular

(Grande and Bemis, 1998; Xu et al., 2014; Xu and Shen, 2015; Sun et al., 2017; Ma and Xu, 2017; Xu and Ma, 2018)

1. Absent
2. Present
3. Posterior margin of median gular

(Grande and Bemis, 1998; Xu et al., 2014; Xu and Shen, 2015; Sun et al., 2017; Ma and Xu, 2017; Xu and Ma, 2018)

1. Smooth
2. Deeply scalloped with a series of sharp points and concavities
3. Number of branchiostegal rays

(Modified from Grande and Bemis, 1998; Xu et al., 2014a,b, 2018, 2019; Xu and Shen, 2015; Sun et al., 2017; Ma and Xu, 2017; Xu and Ma, 2018; López-Arbarello and Sferco, 2018)

1. 10 to 20
2. 9 or fewer
3. 21 or more

**Vertebrate and caudal skeleton**

1. Solid, perichordally ossified, diplospondylous centra in adult-sized individuals

(Grande and Bemis, 1998; Grande, 2010; Cavin et al., 2013; Xu et al., 2014b, 2018, 2019; Xu and Shen, 2015; Deesri et al., 2016; Ma and Xu, 2017; Xu and Ma, 2018; Ebert, 2018)

1. Absent
2. Present
3. Anteriorly projecting spine-like processes on neural and/or haemal arches

(Grande and Bemis, 1998; Xu et al., 2014b, 2018, 2019; Xu and Shen, 2015; Sun et al., 2017; Ma and Xu, 2017; Xu and Ma, 2018)

1. Absent
2. Present
3. Solid vertebral centra of adult-sized individuals

(Modified from Grande and Bemis, 1998; Xu et al., 2014b, 2018, 2019; Xu and Shen, 2015; Sun et al., 2017; Ma and Xu, 2017; Xu and Ma, 2018; Ebert, 2018)

1. Absent
2. Present
3. Surface of solid vertebral centra

(Modified from Grande and Bemis, 1998; Ma and Xu, 2017; Xu and Ma, 2018; Xu et al., 2018, 2019)

1. Smooth
2. Two or more lateral fossae on each side of most centra
3. Number of supraneurals

(Grande and Bemis, 1998; Xu et al., 2014b, 2018, 2019; Xu and Shen, 2015; Sun et al., 2017; Ma and Xu, 2017; Xu and Ma, 2018)

1. 13 or more
2. 5 to 11
3. Opisthocoelous vertebrae

(Xu et al., 2018, 2019)

- - 1. Absent
    2. Present

1. Hypural-ural centra fusion in adult-sized individuals

(Grande and Bemis, 1998; Xu et al., 2014b, 2018, 2019; Xu and Shen, 2015; Sun et al., 2017; Ma and Xu, 2017; Xu and Ma, 2018)

1. All hypurals autogenous (separate) from the ural centra
2. All but first hypural fused to corresponding centra
3. Large parapophyses fused to most of the abdominal centra

(Grande and Bemis, 1998; Xu et al., 2014b, 2018, 2019; Xu and Shen, 2015; Sun et al., 2017; Ma and Xu, 2017; Xu and Ma, 2018; López-Arbarello and Sferco, 2018)

1. Absent
2. Present
3. Number of ural centra

(Grande and Bemis, 1998; Xu et al., 2014; Xu and Shen, 2015; Sun et al., 2017; Ma and Xu, 2017; Xu and Ma, 2018)

1. 10 or fewer
2. 11 to 22
3. Number of preural vertebral centra

(Grande and Bemis, 1998; Xu et al., 2014b, 2018, 2019; Xu and Shen, 2015; Sun et al., 2017; Ma and Xu, 2017; Xu and Ma, 2018)

1. 40 to 73
2. 75 to 82
3. Morphology of pleural ribs

(Grande and Bemis, 1998; Xu et al., 2014; Xu and Shen, 2015; Sun et al., 2017; Ma and Xu, 2017; Xu and Ma, 2018; López-Arbarello and Sferco, 2018)

1. Distal ends pointed or with rounded points
2. Distal ends flatly truncated, even in large adults
3. One-to-one arrangement of hypurals and caudal fin rays

(Grande and Bemis, 1998; Brito and Alvarado-Ortega, 2013; Xu et al., 2014b, 2018, 2019; Xu and Shen, 2015; Sun et al., 2017; Ma and Xu, 2017; Xu and Ma, 2018; López-Arbarello and Sferco, 2018)

1. Last few hypurals each articulate with bases of several caudal fin rays
2. Each hypural normally bears a single caudal ray
3. Number of ossified ural neural arches in adult-sized individuals

(Grande and Bemis, 1998; Brito and Alvarado-Ortega, 2013; Xu et al., 2014b, 2018, 2019; Xu and Shen, 2015; Sun et al., 2017; Ma and Xu, 2017; Xu and Ma, 2018; López-Arbarello and Sferco, 2018)

1. Normally four or more
2. Normally 2 or fewer
3. Numerous paired, block-like ural neural arch ossifications

(Grande and Bemis, 1998; Xu et al., 2014b, 2018, 2019; Xu and Shen, 2015; Sun et al., 2017; Ma and Xu, 2017; Xu and Ma, 2018)

1. Absent
2. Present
3. Long epineural intermuscular bones

(Grande, 2010; Cavin et al., 2013; Deesri et al., 2016; López-Arbarello and Sferco, 2018; Xu et al., 2018, 2019)

1. Absent
2. Present
3. Articulation of proximal end of pleural ribs with vertebral parapophyses

(Modified from Grande, 2010; Xu et al., 2018, 2019)

1. Posterior to parapophyses
2. Anteroventral to parapophyses
3. Shape of haemal spines

(Grande and Bemis, 1998; Xu et al., 2014b, 2018, 2019; Xu and Shen, 2015; Sun et al., 2017; Ma and Xu, 2017; Xu and Ma, 2018; López-Arbarello and Sferco, 2018)

1. Spine-like or rod-like
2. Broadly spatulate in the transverse plane
3. Orientation of preural haemal and neural spines near caudal peduncle

(Grande and Bemis, 1998; Xu et al., 2014b, 2018, 2019; Xu and Shen, 2015; Sun et al., 2017; Ma and Xu, 2017; Xu and Ma, 2018)

1. Positioned at about 25º to 45º from the horizontal
2. Strongly inclined to nearly horizontal
3. Uroneurals

(Modified from Arratia, 2013; Xu et al., 2014b, 2018, 2019; Xu and Shen, 2015; Xu and Ma, 2016, 2018; Sun et al., 2017; Ma and Xu, 2017; López-Arbarello and Sferco, 2018)

1. Absent
2. Present, both preural and ural neural arches modified as uroneurals
3. Present, only ural neural arches modified as uroneurals
4. A gap between hypurals 2 and 3

(Arratia, 2013; Xu et al., 2014b, 2018, 2019; Xu and Shen, 2015; Sun et al., 2017; Ma and Xu, 2017; Xu and Ma, 2018; López-Arbarello and Sferco, 2018)

1. Absent
2. Present
3. Number of epurals

(Grande and Bemis, 1998; Xu et al., 2014; Xu and Shen, 2015; Sun et al., 2017; Ma and Xu, 2017; Xu and Ma, 2018; López-Arbarello and Sferco, 2018)

1. 2-8
2. 10-15

**Girdles**

1. Anteroventral process of posttemporal bone

(Grande, 2010; Cavin et al., 2013; Deesri et al., 2016; Xu et al., 2018, 2019)

- - 1. Absent
    2. Weakly developed
    3. Well developed as a ventral rod-like process

1. Posttemporal penetration by lateral line canal

(Grande, 2010; López-Arbarello, 2012; Cavin et al., 2013; Deesri et al., 2014; López-Arbarello and Sferco, 2018; Xu et al., 2018, 2019)

- - 1. Present
    2. Absent

1. Lateral edge of posttemporal in adult-sized individuals

(Grande and Bemis, 1998; Xu et al., 2014; Xu and Shen, 2015; Sun et al., 2017; Ma and Xu, 2017; Xu and Ma, 2018)

1. Shorter than length of anterior edge
2. Elongate, about equal to or greater than width of anterior edge
3. Posttemporal

(Modified from López-Arbarello and Sferco, 2018; Xu et al., 2018, 2019)

- - 1. Broad (reaching the midline), not contacting parietal
    2. Broad, contacting parietal
    3. Relatively narrow, not reaching the midline (separated by scales)
    4. Much narrow, narrower than the dermopterotic

1. Posterodorsal margin of the supracleithrum peculiarly ornamented

(López-Arbarello and Sferco, 2018; Xu et al., 2018, 2019)

- - 1. Absent
    2. Present

1. Supracleithrum with a concave articular facet for articulation with the posttemporal

(Grande, 2010; López-Arbarello and Sferco, 2018; Xu et al., 2018, 2019)

1. Absent
2. Present
3. Length of anterior arm of cleithrum relative to depth of its dorsal arm

(Xu and Ma, 2018; Xu et al., 2018, 2019)

1. Anterior arm shorter than or nearly equal to dorsal arm
2. Anterior arm notably longer than dorsal arm
3. Substantial scapulocoracoid ossification in adult-sized individuals

(Grande and Bemis, 1998; Xu et al., 2014b, 2018, 2019; Xu and Shen, 2015; Sun et al., 2017; Ma and Xu, 2017; Xu and Ma, 2018)

1. One or more elements present in the shoulder girdle
2. Absent
3. Medial processes of suprascapula

(Grande, 2010; Cavin et al., 2013; Deesri et al., 2016; Xu et al., 2018, 2019)

1. Absent
2. Present
3. Clavicle anterior to cleithrum

(Grande, 2010; Cavin et al., 2013; Deesri et al., 2016; Xu and Ma, 2018; López-Arbarello and Sferco, 2018; Xu et al., 2018, 2019)

1. Present
2. Absent
3. Anterior and posterior ‘clavicle elements’

(Grande, 2010; Cavin et al., 2013; Deesri et al., 2016; Xu and Ma, 2018; López-Arbarello and Sferco, 2018; Xu et al., 2018, 2019)

1. Absent
2. Present
3. Medial wing on cleithrum

(Cavin and Suteethorn, 2006; Grande, 2010; Cavin et al., 2013; Deesri et al., 2016; Giles et al., 2017; Xu et al., 2018, 2019)

1. Present
2. Absent
3. Presupracleithrum

(Gardiner et al., 2005; Xu et al., 2014b, 2018, 2019; Giles et al., 2017)

1. Present
2. Absent
3. Shape of basipterygium

(Grande and Bemis, 1998; Xu et al., 2014; Xu and Shen, 2015; Sun et al., 2017; Ma and Xu, 2017; Xu and Ma, 2018)

1. Proximal end flat and widened anteriorly
2. Proximal end long and rod-like, without significant widening anteriorly
3. Pectoral propterygium fused with first ray

(Arratia, 2013; Xu et al., 2018, 2019)

1. Absent
2. Present

**Fins**

1. Shape of posterior margin of caudal fin

(Grande and Bemis, 1998; Brito and Alvarado-Ortega, 2013; Xu et al., 2014b, 2018, 2019; Xu and Shen, 2015; Sun et al., 2017; Ma and Xu, 2017; Xu and Ma, 2018)

1. Forked
2. Convexly rounded or nearly vertical
3. Fringing fulcra on pectoral fins

(Xu et al., 2018, 2019)

- - 1. Present
    2. Absent

1. Fringing fulcra on pelvic fins

(Xu et al., 2018, 2019)

- - 1. Present
    2. Absent

1. Fringing fulcra on median fins

(Grande and Bemis, 1998; Xu et al., 2014b, 2018, 2019; Xu and Shen, 2015; Sun et al., 2017; Ma and Xu, 2017; Xu and Ma, 2018)

1. Present
2. Absent
3. Number of dorsal fin(s)

(Ebert, 2018; Xu et al., 2018, 2019)

- - 1. Single
    2. Two

1. Shape of (first) dorsal fin

(Gardiner and Schaeffer, 1989; Xu et al., 2018, 2019)

- - 1. Triangular
    2. Bow-shaped

1. Origin of (first) dorsal fin

(López-Arbarello, 2012; Xu et al., 2018, 2019)

- - 1. Posterior to the origins of pelvic fins
    2. Opposite or anterior to the origins of pelvic fins

1. Number of dorsal fin rays

(López-Arbarello, 2012; Deesri et al., 2016; Xu et al., 2018, 2019)

- - 1. 20 or more
    2. Less than 20

1. Dorsal and anal fin rays

(Modified from Gardiner et al., 1996; Xu and Gao, 2011; Xu and Wu, 2012; Xu et al., 2012, 2014a,b, 2015, 2018, 2019)

1. rays more numerous than radials
2. rays and radials largely equal in number
3. Number of principal caudal fin rays below the lateral line in adults

(López-Arbarello, 2012; López-Arbarello and Wencker, 2016; Xu et al., 2018, 2019)

- - 1. Nine or more
    2. Eight or seven
    3. Six or less

1. Predorsal length

(Xu et al., 2018, 2019)

- - 1. 70% or less of SL
    2. 75% or more of SL

1. Enlarged dorsal scute (corresponding to more than one vertical row of scale) preceding caudal fin

(Arratia, 2013; Xu et al., 2018, 2019)

1. Absent
2. Present
3. Number of epaxial procurrent caudal fin rays

(Grande and Bemis, 1998; Xu et al., 2014b, 2018, 2019; Xu and Shen, 2015; Sun et al., 2017; Ma and Xu, 2017; Xu and Ma, 2018)

1. 0 to 11
2. 12 to 15
3. Lateral line ossicles extending onto caudal fin

(Gardiner et al., 1996; Xu et al., 2014b, 2018, 2019; Xu and Shen, 2015; Sun et al., 2017; Ma and Xu, 2017; Xu and Ma, 2018; López-Arbarello and Sferco, 2018)

1. Absent
2. Present

**Body shape and scales**

1. An apparent dorsal hump between head and dorsal fin

(Xu and Wu, 2012; Xu et al., 2018, 2019)

1. Absent
2. Present
3. Scales

(Alvarado-Ortega and Espinosa-Arrubarrena, 2008; Brito and Alvarado-Ortega, 2013; Xu et al., 2012, 2014b, 2018, 2019; Xu and Shen, 2015; Sun et al., 2017; Ma and Xu, 2017; Xu and Ma, 2018; Ebert, 2018)

1. Rhomboid
2. Amioid-type, subrectangular to elongate oval
3. Elasmoid of cycloid type
4. Dorsal peg of rhomboid scale

(Modified from López-Arbarello, 2012; Deesri et al., 2016; Xu et al., 2018, 2019)

- - 1. Present
    2. Absent

1. Anteroventral articular process of rhomboid scale

(Xu et al., 2018, 2019)

- - 1. Absent
    2. Present

1. Spines on dorsal ridge scales anterior to dorsal fin

(Olsen and McCune, 1991; Grande, 2010; Xu and Wu, 2012; López-Arbarello, 2012; Xu et al., 2018, 2019)

- - 1. Absent
    2. Present

1. Flank scales with large prominent posteriorly pointing spines

(Grande, 2010; López-Arbarello, 2012; Cavin et al., 2013; Deesri et al., 2016; Xu et al., 2018, 2019)

- - 1. Absent
    2. Present

1. Complete row of elongated scales between last lateral line scale and uppermost caudal fin ray

(López-Arbarello, 2012; López-Arbarello and Wencker, 2016; López-Arbarello and Sferco, 2018; Xu et al., 2018, 2019)

- - 1. Absent
    2. present

1. Urodermals in the caudal

(Grande and Bemis, 1998; Xu et al., 2014b, 2018, 2019; Xu and Shen, 2015; Sun et al., 2017; Ma and Xu, 2017; Xu and Ma, 2018)

1. Present
2. Absent

Part C Data matrix of taxa and characters

Pteronisculus_stensioi_

0----0----00-00-0000?0000000000010000000000000---000----00000000000-00000-001000??0000000000000-000?000000-00000000-0-0-00000---00000000000000000000000000000-0--00000000-00?0???0000-0000?0000000000000?00000000000000000000000

Teffichthys_madagascariensis

100?0?00??0100000000?0000000000000000000000001000000----100000000000000000?00000??00???0??0000??01-?000?00-00000000-0-0-00010---0000????000?0000?000000000000-0--000010?0-?0?0???0?????????0000000???000??0000000110000000000000

Watsonulus_eugnathoides

1?0?0000?002000?100000200000000000001110000011101000----10100110000000000100210000000000??0000110120100?01100010101010000001100010000000000000000001003000101010010000000-00000000?00?0000020000?0000000?00000000110000000000000

Amia_calva

1000101100020001100100201001000000001111010010---010----00101110000-00010111-1--000000000001001101-010001110001010101000100-1000100000000000000010000010000010100100001?10101101011001000002000001101111001111011010000101--0001

Cyclurus_kehreri

?0001?11?012000?1?01?0200000000000001111000010---010----00101120000-00010111-1--0?0000000001001101-0100?1110001010101000100-1000100010000000000010000010000010100100001110101100011001000002000001101?11001111011011000001--0001

Vidalamia_catalunica

??????1??002?00?1????0201000101000001110010011000?10----00101110000000010101-1--0?0000000001011101-0100??110001010111000000-10101000000000000000?0001011100010100100001111?00010111001000012010001001?1110111101101?000001--0000

Calamopleurus_cylindricus

1(01)0(01)101110020001100100201000000000011210010011000100----00100120000000000101-1--000000000001011101-011001110001010101000000-1010100001100000000010001010000010100100101110000010011001000002010001001?11001111001111000101--0000

Pachyamia_mexicana

?????????002?00?1??1?0201000101000001110010111001100----001011100000?1010101-1--0?0000000001011101-0100?1110001010111000000-1011100000000000000010001011100010100101001110?0001111100100001?010001001?1110111101101?001001--0000

Amiopsis_lepidota

?????????00200011????0200000000000001110000011001100----0000011000000001010001000?0000000001001101-0100??11000101010100010011010100000000000000000000010000010100100001111000000011001000002000001001?11001110001110000001--0000

Ikechaoamia_meridionalis

???????????2?00?1????020-10000012000111101001100?11?----0000011000000001?1?1-1--0???0?00??01001101-01????110001010101000100-1010100000?000000?00?000001000000-10010??0001100000001100?00000?0000010?1???001111011012000000100000

Sinamia_zdanskyi

??0?1?11?0?210011000?020-100000120001111010011000100----00100110000000010111-1--0?000000??01001101-0100?1110001010101000100-101010000??0000?0?00?000101000000-1001000000110000000??00?00000?010001001?11?01111011012000000100000

Solnhofenamia_elongata

???????1?002?00?1?01?0200000000000001110000011001100----0000011000000001010001000?0000000001001101-0100??1100010101010001001101010000000000000000000001?100010100100001110000000011001000002000001001?11001111011010001001--0000

Liodesmus_gracilis

???????????2?00?1????020000000000000111000001100?100----00?00110000000010100?1000???0??0??01001101-0100??11000111010100000011010100000?000??0?00?001101000001010010002000-0?00000010??11000?0000?1??1?1?0?0000001110000001--00?0

Oshunia_brevis

10001001100210011000002010000100000011110?0010---??0----01010110000-101101?021000????000?001001101201???0110001011101?00100110101000000000??0?00000?001000001010010?0?00110000?00000??0000020000?0001???0?0?0000001000?001--0000

Teoichthys_kallistos

????????????????1????02010000101000011100?00110011?0----001101101111101101?02100???????0??0000??01-???????????1011101000100110101000???000??0?00?001001000001010010??01?100????0????????00??0020?00???????0000001010000?00000000

Macrepistius_arenatus

10001000?02210011000002010000101000011100?0011001110----0?1101101111101101?02100?????0?0??0000??01-???????????101?101000100110101000?0?000??0?00?0000010000010100100?01010???0?0??????0000??0020?0??1?????0??0001010000100000000

Ophiopsis_muensteri

????????????????1?0?00201000010100001110000011001110----000101100000?011?1?011000?000??0??00001?01?01??0?110001011101?00100110101000??00000?0?00?001001000101010010000??10??????????????????002010?01????00?00000110000000000000

Cerinichthys_koelblae

??????????????????0?002000000100000011100??011001??0----100101101000?0?1?1?01101???????0???0001?01?01????1100?101?10100000011010100000?0000???00?00??01000001010010000000-0-?0?00???0????????000??????????0000001110000000??0000

Heterolepidotus_latus_

10001000?0020001?00?00200?0001?0000011100??011?0?110----0011011?0001?????1?011100?000?00??00001101?0100??11000101?10100010011010100000?0000??00000010010000010100???????1????????????????????000????????0000000011?00?0?00??0000

Panxianichthys_imparilis

????????????????1????02010000000000011100?0011101100----000002110000000001001100???????0??01001?00101????110001011101000100110001000???000??0?00?0010010000010100100?0??0-??????????????????000010?????0??0000000110000000000000

Subortichthys_triassicus

??????????0?????1???00200000000020001110000011001100----(01)00001110000000001001100??0?0??0???101110010100??11000101110100200011000100000?000??0?00?001001000001010010000??0-??????????????????000010???????00000000110000000000010

Caturus_furcatus

10001011?0020001100000200000000000001110010011001100----000001100000000101001100000000000001001101?0100011100011101010001001101010000?10000000000001101000001010010002000-00000000010?110002000011001110000000001110000001--00?0

Quetzalichthys_perrilliatae

????????????????1????02010000100000011100?0011?01110----011101110001101101?01100???????0??00001?01?0100??110001011101?0000011010100000?000??0?00?000001000001010010?000?110??0000000??00000?0000100?1?????0?00000010000001--00?0

Ionoscopus_cyprinoides

12001000100210011001002010000000000011100?00110010?0----0110011100011011010011000?0?0000000100110120100??1100010111010000001101010000010000?0000000000100010101001000000110000?00000?1000002000010001???000000000110000001--0000

Ophiopsiella_procera

?????????????00?1????0200000010100001110000011000110----0011011010111011010011000????0?0??00001?01-01????110001011101000100110101000?0?000??0?00?000001000001010010?001?100?000000?0??00000?0000?00?1???000000001010000100000000

Congophiopsis_lepersonnei

????????????????1???002010000101000011100?00110011?0----00110??01011?01?0??01100???????0???0001?01-01????11000101110100010011010100????000????00?0010010000??01001??????10???????????????????000?0??1?????010000111?010?00?00000

Robustichthys_luopingensis

?????????002?00?1???00201000010000001110000011101110----0001011100001010010021000?000?00??0010110010100?01100010110010001001100010000000000?0000?001001000001010010000??0-?0???????????????2000010??1100?00000001010000000000000

Asialepidotus_shingyiensis

?????????002?00?1???00201000010020001110000011101100----0000011100001010010011000?000?00??0100110010100001100010111010001001100010000000000?0000?001001000001010010000??0-?0????????????????000010??1100?00000000110000000000000

Elops_hawaiensis_

100010100002010000001030000001000000-311000001201000----10100111000000000011-1--100000001011011001-0000100-00010100-1202010-11-01001--0101-1010-0021002000000-10011--20011000100000010002102000000001011010111001110010002--0000

Leptolepis_coryphaenoides

100010100002011000001030000001000000-310000001100000----100001110000000000?001001?0000001011011011-0001?00-00010100-1102110111-01001--?111-1001-0001002001100-100100000010?000000000100?2102000000001011?10111001110010002--0001

Dorsetichthys_bechei

1000100000020110000010100000000000000200000001100000----1000011100000000000001001?0000001011011001-0000?00-00010100-1002110111-0?00000?0110100000001002000111010010000000-?0000000001000110?00000000??1??10000000110010000000000

Ichthyokentema_purbeckensis

1101101000020100000010101000000000001110000001001000----100001100000000000000100100?0000??11001001-0000?00-00010100-1100110110001000000111?10?0-?001002000001010010000??10?0??????????????020000?0??1?11??0000000110010000000000

Pholidoctenus_serianus

????????????????0???0010-----0--000011000-0001100000----1000011100000000?1001100??0?0??0???000??01-?0???00-0001010(01)?1?02110111-0100????011??0?00?001002000101010010000??0-?0????????0?00?1??0000?0????10?00000000110010000000010

Pliodetes_nigeriensis

?????????????????????02010000111100011100100111000010?1100100?2011010000?0?021000?????00???000??10???00?11111010100-1?0110?10---110???0100??0?11?0?0?12000001000-11--???1??000?????????????00020?0??1?????000000011?00?0000001?0

Tlayuamichin_itztli

?????????0??????1???0020100001111000111000?0110000012000001002211100000000?011000???0?00???100??2?100????1100010100-1000201110101100110000??0?100001001000101110011--1?????0???????????????0002010??1??1?00000000111000000011000

Eosemionotus_diskosomus

?????????0?2?????????0200010-111200?111101100----00111-00010022?000-???????1-1--00??0?00??0100??101?00??01000010100-1000201-0---11?00??0000???10?001012000101010111--1??????????????????????0000?0??1?????0?000001110000000?1010

Macrosemius_rostratus

?11--110?0?2-??000000020100001120110111?01001????00121-00010022?010000000011-1--0?000000000100?02010000?01000010100-1?00200-0---100010?00001001?0000002000111010111--1??0-?0?1??????0???????001010?01?0100111101101100000000-0?0

Notagogus_denticulatus

????????????????????0020?000011201101?100100110-0001??-00010022001000?000011-1--??????00???100??101?000??1100010100-1?00200-0---100????0000?0?1??000002000111010111--1?????0????????0???????001010??1??1?101001010110000000000?0

Propterus_elongatus

?????1??????????0???00201000011201101110010011000001210000100220010000000011-1--0?????00???1001?201?000??1100010100-1?00200-0---100????0000?0?1??000002000011010111--1??0-10???0???????0?0??001010??11?1?00100101011000000000000

Kyphosichthys_grandei

?????????????????????02010000112010011100000111000010100101002111100000000001100???????0??0010??001?0????1100010100-10001001100010000??000??0?(01)0?0010010001010100100010?0-???0??????????????001010?????1?00000000110000010001010

Macrosemimimus_fegerti

?????0???1??????1????02010000111010011100000110000012000001002200100000000001100???0??00???100??101?0???11100010100-1000200110101100100000???010?001001000101110011--1??????????????????????002110??1??1?000000001110000000?1000

Semionotus_elegans

?11--?1???02-00?100?0020(01)00001110000111000001100000102110010022001010000?0?0010000??0000000100101010000011100010100-10002001101010000000000100100001001000001010011--1000-00000000000?000002002010?01101000000000111000000001000

Lepisosteus_osseus

011--1110102-000010100201000111120001110111011110001202110100220110100001010200001111111010010122011000011111010000-101----10---1-10001000011001001000201100100--11--10011010100010001000001103000011101001000000112100000000000

Atractosteus_spatula

0(01)1--1110102-000010100201000011120001110111011110001202110100220110100001010200001111111010010122011000011111010000-101----10---1-10001000110001001000201100110--11--10011010100010001000001103000011101001000000112100000100000

Obaichthys_decoratus

?1???1110102?00011110121100001112000111011101100000120111010022011010?00?01021000??01110010100??201?0000?1111110100-10012001????100000?0000100110000012010001010111--10011?1010?0100010?000??030000000010010000001121000000001?0

Dentilepisosteus_laevis

?101?1110102000011010121100001111000111011101101000120211010022011010?00?010210001101110010100??201?000?11111110100-100120011010100000?00001001?0000012010001010111--10011?1010001000?0000020030?0001?01??1000000112100000000100

Masillosteus_janeae

??1--?11?102-00?0?010020100001111000111011101111000121211010022011010(01)0010102000????1?10??0010??201?000?1111?0?0?00-101----10---1-1010?000010011000001200100100--11--10011?1010001000?000001103000??1?01??1000000112100000000000

Cuneatus_wileyi

0?????110102?00???0?00201000011120001110?11011(01)100012021101002201101000010102000??111110??0010??201?000?111110?0?00-101----10---1-1000?000010011000000200100110--11--10011?1010001000?0000011030000?1?01??10000001121000000?0000

Araripelepidotes_temnurus

11???0100022?00?000000201001001110001?1001?01110000101?010100?21110?0000?0?01100??????00??00101?1??1???????0??10100-0-0110010---11??????00??0?11?1-0?12000001010111--???0-?0????????????????002000??1????00000000111000000010100

Yudaiichthys_eximius

??????????2?????????0020100000120100111100001120000102000010022101000000?0?01010??????00???000??001??????1100010100-100010010---100000?000????00?001001000101011010001??????????????????????0010?0?????1??0000000111000000001010

Fuyuanichthys_wangi

??????????2?????????00201000011201001111000010---00101-010100210110-0000?0?01100??????00???010??101?0????1100010100-100010010---110?0??000????10?001011000101010010001??0-?0????????????????0010?0?????1??000000011(01)000000001010

Lashanichthys_sui

?????????022?00?1???00201000001201001110000011(01)0000102001010022101000000000011110?000?00?00000100010000011100010100-10001001100010000000000?0000?00100100000101101(01)001000-00-0???0??0??000?2001010??1101?00000000110000000001010

Lepidotes_semiserratus

1101?0??0???0??010010020100001110000111000?0111000011121001002210101000000?01110??????00??00001010100????110?010100-1?00200110101000???000??0?1??001001000001010011--1??0-?0????????????????00?010??1??1??0000000?1000?0000100?0

Thaiichthys_buddhabutrensis

1(01)01?11??12200001?00002010000111200011100110111100011121101002210101000010?011000?00??000?0010101011000?11100010100-1?0120010---100???0000??0010?001002000001010?11--??00-00???0???????????00020?0??1?0?0?000000011000?0000110?0

Ticinolepis_longaeva

?????????0??000?????00200000010000001110010011(01)000010000001002100000000000?0211000?????0??0000100010000?01100010100-10001001100010000000000??010?001001000001010011--1??0-?0????????????????000010????00??0000000110000000000000

Semiolepis_brembanus

????????????????????00201000011120001?100??0110000011000101002201100000000?00100???0??00???100??1?????0??1100010100-1?00200110?0100010??00??0?1??001001000001010111--??????0????????????????002010????????0000000110000000001000

Isanichthys_palustris

?????????1??????0????0201000010110001?1000?0?1110001??210010022111010100?0?02110???????????000??1?????????????10100-100020011010100????000??0?10?0?1001000001010011--1??0-?0????????????????0020??????????0??0000?1?000000??00?0

Sangiorgioichthys_aldae

????????????????????0020100001(01)0000011100000111000010100(01)0100?1011010000?0?02111???????????000??1????????1100010100-10002001101011?????000??0?10?0?1011000101010011--?0?0-??????????????????0000?0?????1??00?000011?000000001010

Sangiorgioichthys_valmarensis

????????????????????002010000100001011100000111???????0?1?????101???0000?0?01111???????0?0?000??1???????????0010100-??0?2001??10100?????01??0?1??00?0110000010?0011--10?????????????????????000010????01??00?0001110000000001010

Lophionotus_chinleana

????????????????????00201000011110001?1000?011100001?2?11010022001010000?0?00100??????00???100??101?0????1100010100-10002001??1?10?????000??0?1??001001000001010011--???????????????????????0000?0?????0??0000000110000000001000


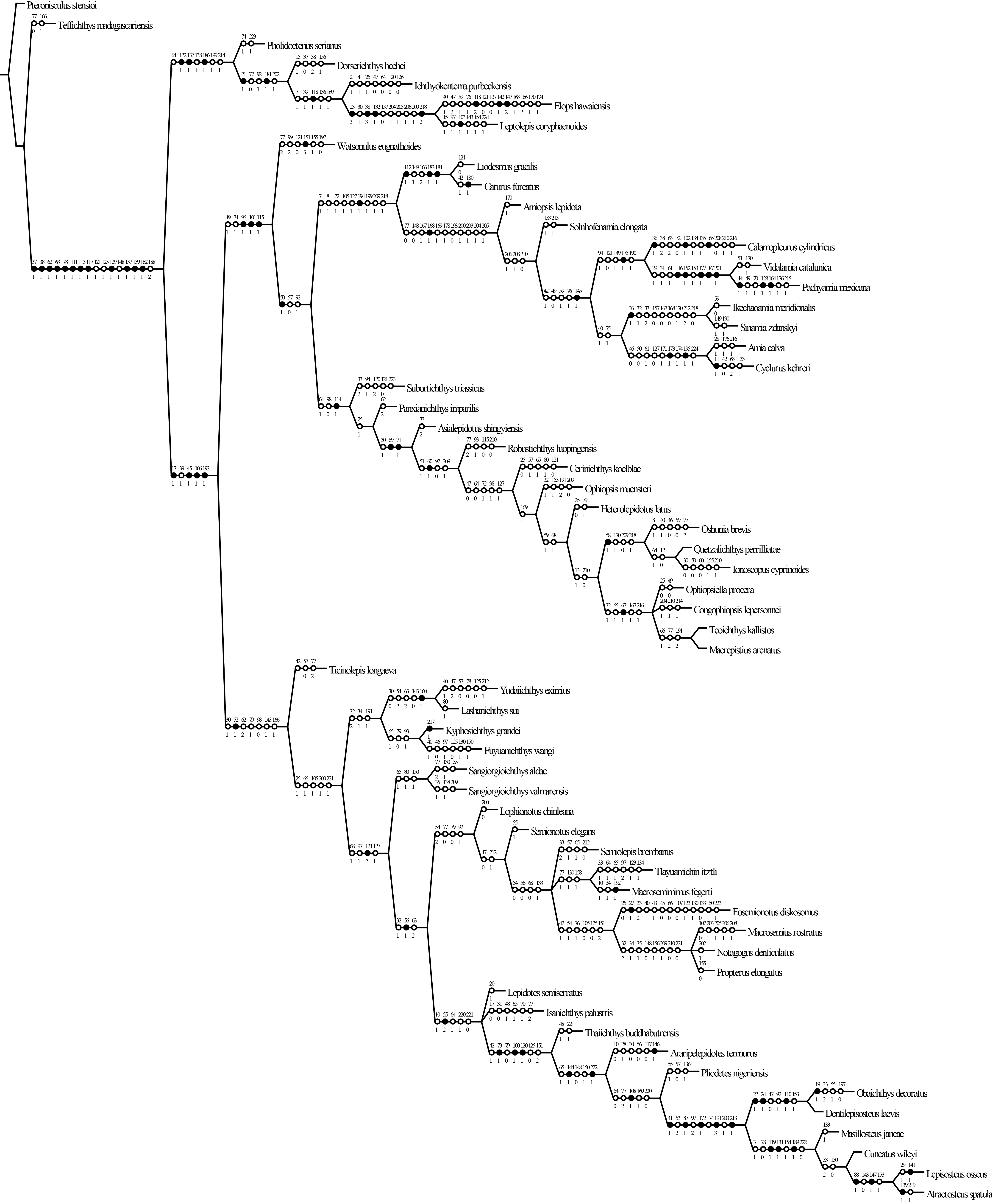
Part D Supplementary figure

**Fig. 1S** Strict consensus of 24 most parsimonious trees (tree length = 650 steps, consistency index = 0.4185, retention index = 0.7680), illustrating the phylogenetic relationships of *Robustichthys* within the Neopterygii. Character changes indicated with solid circles are unique.

Part E Literature cited

Alvarado-Ortega J, Espinosa-Arrubarrena L, 2008. A new genus of ionoscopiform fish (Halecomorphi) from the Lower Cretaceous (Albian) lithographic limestones of the Tlayúa quarry, Puebla, México. J Paleont, 82: 163–175.

Applegate SP, 1988. A new genus and species of a holostean belonging to the family Ophiopsidae, *Teoichthys kallistos*, from the Cretaceous near Tepexi de Rodríguez, Puebla. Inst Geol, Rev, 7: 200–205.

Arratia G, 2013. Morphology, taxonomy, and phylogeny of Triassic pholidophorid fishes (Actinopterygii, Teleostei). Soc Vert Paleont Mem (Suppl J Vert Paleont), 13: 1–138.

Bartram AWH, 1977. The Macrosemiidae, a Mesozoic family of holostean fishes. Bull Br Mus Geol, 29: 137–234

Bartram AWH. 1975. The holostean fish genus *Ophiopsis* Agassiz. Zool J Linn Soc, 56: 183–205.

Brito PM, Alvarado-Ortega J, 2013. *Cipactlichthys scutatus*, gen. nov., sp. nov. a new halecomorph (Neopterygii, Holostei) from the Lower Cretaceous Tlayúa Formation of Mexico. PLoS ONE, 8: e73551.

Cavin L, 2010. Diversity of Mesozoic semionotiform fishes and the origin of gars (Lepisosteidae). Naturwissenschaften, 97: 1035–1040.

Cavin L, Deesri U, Suteethorn V, 2013. Osteology and relationships of *Thaiichthys* nov. gen.: a Ginglymodi from the Late Jurassic–Early Cretaceous of Thailand. Palaeontology, 56: 183–208.

Cavin L, Suteethorn V, 2006. A new Semionotiform (Actinopterygii, Neopterygii) from Upper Jurassic–Lower Cretaceous deposits of North-East Thailand, with comments on the relationships of Semionotiforms. Palaeontology, 49: 339–353.

Chen W-Q, Sun Z-Y, Tintori A et al., 2014. A new species of *Sangiorgioichthys* Tintori& Lombardo, 2007 (Actinopterygii; Semionotiformes) from the Pelsonian (Anisian, Middle Triassic) of Guizhou Province, South China. Neu Jahrbuch für Geol Paläont, Abha, 273: 65–74.

Coates MI, 1999. Endocranial preservation of a Carboniferous actinopterygian from Lancashire, UK, and the interrelationships of primitive actinopterygians. Philos Trans R Soc London B, 354: 435–462.

Deesri U, Jintasakul P, Cavin L, 2016. A new Ginglymodi (Actinopterygii, Holostei) from the Late Jurassic–Early Cretaceous of Thailand, with comments on the early diversification of Lepisosteiformes in Southeast Asia. J Vert Paleont, 36: e1225747.

Deesri U, Lauprasert K, Suteethorn V et al., 2014. A new species of the ginglymodian fish *Isanichthys* from the Late Jurassic Phu Kradung Formation, northeastern Thailand. Acta Palaeont Pol, 59: 313–331.

Ebert M, 2018. *Cerinichthys koelblae*, gen. et sp. nov., from the Upper Jurassic of Cerin, France, and its phylogenetic setting, leading to a reassessment of the phylogenetic relationships of Halecomorphi (Actinopterygii). J Vert Paleont, 38: e1420071.

Forey PL. 1973. A revision of the elopiform fishes, fossil and recent. Bull Brit Mus(Nat Hist), Geol, 10 (Supp): 1–222.

Gardiner BG, Maisey JG, Littlewood DTJ. 1996. Interrelationships of basal neopterygians. In: Stiassney M L J, Parenti L R, Johnson G D eds. Interrelationships of fishes. San Diego: Academic Press. 117–146.

Gardiner BG, Schaeffer B, 1989. Interrelationships of lower actinopterygian fishes. Zool J Linn Soc, 97: 135–187.

Gardiner BG. 1960. A revision of certain actinopterygian and coelacanth fishes, chiefly from the Lower Lias. Bull British Mus (Nat Hist), Geol 4:239–384.

Gibson SZ, 2013. Biodiversity and evolutionary history of †*Lophionotus* (Neopterygii: †Semionotiformes) from the western United States. Copeia, 2013: 582–603.

Giles S, Xu G-H, Near TJ et al., 2017. Early members of ‘living fossil’ lineage imply later origin of modern ray-finned fishes. Nature, 549: 265–268.

Grande L, 2010. An empirical synthetic pattern study of gars (Lepisosteiformes) and closely related species, based mostly on skeletal anatomy. The resurrection of Holostei. Copeia, 10(Suppl): 1–871.

Grande L, Bemis WE, 1998. A comprehensive phylogenetic study of amiid fishes (Amiidae) based on comparative skeletal anatomy: an empirical search for interconnected patterns of natural history. Soc Vert Paleont Mem (Suppl J Vert Paleont), 4: 1–690.

Griffith J, Patterson C. 1963. The structure and relationships of the Jurassic fish *Ichthyokentema purbeckensis*. Bull Brit Mus (Nat Hist ), Geol, 8: 1–43.

Lambers PH. 1992. On the Ichthyofauna of the Solnhofen Lithographic Limestone (Upper Jurassic, Germany). Unpublished D. Phil. Thesis, Rijksuniversiteit Groningen.

Lehman JP. 1952. Étude complémentaire des poissons de l’Eotrias de Madagascar. Kung Sven Vet Hand, 2: 1–201.

Lombardo C, Tintori A, Tona D. 2012. A new species of *Sangiorgioichthys* (Actinopterygii, Semionotiformes) from the Kalkschieferzone of Monte San Giorgio (Middle Triassic; Meride, Canton Ticino, Switzerland). Boll Soc Paleont It, 51: 203–212.

López-Arbarello A, 2012. Phylogenetic interrelationships of ginglymodian fishes (Actinopterygii: Neopterygii). PLoS ONE, 7: e39370.

López-Arbarello A, Alvarado-Ortega J, 2011. New semionotiform (Neopterygii) from the Tlayúa Quarry (Early Cretaceous, Albian), Mexico. Zootaxa, 2749: 1–24.

López-Arbarello A, Sferco E, 2018. Neopterygian phylogeny: the merger assay. R Soc Open Sci, 5: 172337.

López-Arbarello A, Stockar R, Bürgin T, 2014. Phylogenetic relationships of the Triassic *Archaeosemionotus* Deecke (Halecomorphi, Ionoscopiformes) from the ‘Perledo Fauna’. PLoS ONE, 9: e108665.

López-Arbarello A, Wencker LCM, 2016. New callipurbeckiid genus (Ginglymodi: Semionotiformes) from the Tithonian (Late Jurassic) of Canjuers, France. Paläont Z, 90: 543–560.

Ma X-Y, Xu G-H, 2017. A new ionoscopiform fish (Holostei: Halecomorphi) from the Middle Triassic (Anisian) of Yunnan, China. Vert PalAsiat, 55: 92–106.

Marramà G, Lombardo C, Tintori A, Carnevale G. 2017. Redescription of ‘*Perleidus*’ (Osteichthyes, Actinopterygii) from the Early Triassic of northwestern Madagascar. Riv Ital Paleont Str 123: 219–242.

Nielsen E. 1942. Studies on Triassic fishes from East Greenland. I. *Glaucolepis* and *Boreosomus*. Medd Grønland, 138: 1–403.

Olsen PE, 1984. The skull and pectoral girdle of the parasemionotid fish *Watsonulus eugnathoides* from the Early Triassic Sakamena Group of Madagascar, with comments on the relationship of the holostean fishes. J Vert Paleont, 4: 481–499.

Olsen PE, McCune AR, 1991. Morphology of the *Semionotus elegans* species group from the Early Jurassic part of the Newark Supergroup of eastern North America with comments on the family Semionotidae (Neopterygii). J Vert Paleont, 11: 269–292.

Patterson C. 1975. The braincase of pholidophorid and leptolepid fishes, with a review of the actinopterygian braincase. Phil Tran R Soc London (B), 269:275–579.

Schröder KM, López-Arbarello A, Ebert M, 2012. *Macrosemimimus* gen. nov. (Actinopterygii, Semionotiformes) from the Late Jurassic of Germany, England and France. J Vert Paleont, 32: 512–529.

Sun Z-Y, Ni P-G, 2018. Revision of *Kyphosichthys grandei* Xu & Wu, 2012 from the Middle Triassic of Yunnan Province, South China: implications for phylogenetic interrelationships of ginglymodian fishes. J Syst Palaeont, 16: 67–85.

Sun Z-Y, Tintori A, Xu Y-Z et al., 2017. A new non-parasemionotiform order of the Halecomorphi (Neopterygii, Actinopterygii) from the Middle Triassic of Tethys. J Syst Palaeont, 15: 223–240.

Taverne L. 2015. Osteology and phylogenetic relationships of *Congophiopsis lepersonnei* gen. nov. (Halecomorphi, Ionoscopiformes) from the Songa Limestones (Middle Jurassic, Stanleyville Formation), Democratic Republic of Congo. Geo-Eco-Trop, 38:223–240.

Thies D. 1996. The jaws of *Araripelepidotes temnurus* (Agassiz, 1841) (Actinopterygii, Semionotiformes) from the Early Cretaceous of Brazil. J Vert Paleont, 16: 369–373.

Tintori A, Lombardo C, 2007. A new early Semionotidae (Semionotiformes, Actinopterygii) from the Upper Ladinian of Monte San Giorgio area (Southern Switzerland and Northern Italy). Rivi Paleont Str, 113: 369–381

Wenz S, 1999. *Pliodetes nigeriensis*, gen. nov. et sp. nov., a new semionotid fish from the Lower Cretaceous of Gadoufaoua (Niger Republic): phylogenetic comments. In: Arratia G, Schultze H-P, eds. *Mesozoic Fishes 2—Systematics and Fossil Record*. Munich: Verlag Dr. Friedrich Pfeil, 107–120.

Xu G-H, Gao K-Q, 2011. A new scanilepiform from the Lower Triassic of northern Gansu Province, China, and phylogenetic relationships of non-teleostean Actinopterygii. Zool J Linn Soc, 161: 595–612.

Xu G-H, Gao K-Q, Coates MI, 2015. Taxonomic revision of *Plesiofuro mingshuica* from the Lower Triassic of northern Gansu, China, and the relationships of early neopterygian clades. J Vert Paleont, 35: e1001515.

Xu G-H, Gao K-Q, Finarelli JA, 2014a. A revision of the Middle Triassic scanilepiform fish *Fukangichthys longidorsalis* from Xinjiang, China, with comments on the phylogeny of the Actinopteri. J Vert Paleont, 34: 747–759.

Xu G-H, Ma X-Y, 2018. Redescription and phylogenetic reassessment of *Asialepidotus shingyiensis* (Holostei: Halecomorphi) from the Middle Triassic (Ladinian) of China. Zool J Linn Soc, 184: 95–114.

Xu G-H, Ma X-Y, Ren Y, 2018. *Fuyuanichthys wangi* gen. et sp. nov. from the Middle Triassic (Ladinian) of China highlights the early diversification of ginglymodian fishes. PeerJ, 6: e6054.

Xu G-H, Ma X-Y, Wu F-X, Ren Y, 2019. A Middle Triassic kyphosichthyiform from Yunnan, China, and phylogenetic reassessment of early ginglymodians. Vert PalAsiat, DOI: 10.19615/j.cnki.1000-3118.190319.

Xu G-H, Shen C-C, 2015. *Panxianichthys imparilis* gen. et sp. nov., a new ionoscopiform (Halecomorphi) from the Middle Triassic of Guizhou, China. Vert PalAsiat, 53: 1–15.

Xu G-H, Wu F-X, 2012. A deep-bodied ginglymodian fish from the Middle Triassic of eastern Yunnan Province, China, and the phylogeny of lower neopterygians. Chin Sci Bull, 57: 111−118.

Xu G-H, Zhao L-J, 2016. A Middle Triassic stem-neopterygian fish from China shows remarkable secondary sexual characteristics. Sci Bull, 61: 338–344.

Xu G-H, Zhao L-J, Coates MI, 2014b. The oldest ionoscopiform from China sheds new light on the early evolution of halecomorph fishes. Biol Lett, 10: 20140204.

Xu G-H, Zhao L-J, Gao K-Q et al., 2012. A new stem-neopterygian fish from the Middle Triassic of China shows the earliest over-water gliding strategy of the vertebrates. Proc R Soc, Ser B, 280: 20122261.
